# Sleep regulation in *Drosophila*: a review of neural circuits and genetics

**DOI:** 10.3389/fnins.2026.1750211

**Published:** 2026-01-29

**Authors:** Yiyang Zhao, Kexing Zhang, Hongsheng Bian, Xiaoyan Ma, Songlin Wang, Yanyan Wang, Shuang Yu, Lili Huang

**Affiliations:** 1 College of Pharmacy, Heilongjiang University of Chinese Medicine, Harbin, China; 2 Heilongjiang Provincial Hospital, Harbin, China

**Keywords:** *Drosophila*, genes, mechanism, neural circuits, sleep

## Abstract

Sleep in *Drosophila melanogaster* is regulated by a complex and distributed network of neural circuits that are influenced by factors such as internal state, circadian timing, and prior experiences. While no single “sleep center” has been identified, key brain regions—including the central complex, the mushroom bodies, and other associative structures—such as ventral nerve cord (VNC) contribute to the modulation of sleep and wakefulness. The roles of these regions appear to be dynamic, context-dependent, and often overlapping, reflecting the multifaceted nature of sleep regulation. At the circuit level, mechanisms such as changes in neuronal firing patterns, neurotransmitter systems (e.g., octopamine, dopamine, GABA), and experience-dependent synaptic plasticity have been shown to regulate sleep-wake cycles. On a molecular scale, a variety of genes—including *shaker, fruitless*, and *GAT*—influence sleep regulation through distinct pathways, with perturbations in these genes resulting in significant alterations in sleep duration, architecture, and homeostatic regulation. Recent studies, particularly those utilizing *Drosophila* sleep mutants, have provided valuable insights into the genetic and circuit-level interactions that govern sleep homeostasis and its coordination with the circadian system. These findings underscore sleep as an emergent property of interacting neural and genetic networks, providing a robust model for understanding the mechanisms of sleep in more complex organisms. This review synthesizes the latest advancements in *Drosophila* sleep research, with a focus on neural structures and the genetic basis of sleep regulation.

## 1 Introduction

Sleep represents a fundamental state in life processes, essential for maintaining physical health and normal physiological functions (Ramar et al., 2021). Research has established that sleep permeates bodily metabolism, energy regulation, immune modulation, and cognitive learning (Vaccaro et al., 2020; Titos et al., 2023; Xie et al., 2013; Du et al., 2024; Sang et al., 2023; Geva-Sagiv et al., 2023) Sleep deprivation manifests as fatigue, impaired attention, emotional dysregulation, compromised judgment, and reduced physical coordination. Chronic sleep deficiency increases risks of diabetes, obesity, hypertension, cardiovascular disease, and depression (Taheri et al., 2004; Ren et al., 2023; McAlpine et al., 2019; Chen et al., 2012; Robertson et al., 2013).

The two-process model of sleep regulation, proposed in 1982 and widely accepted in sleep medicine, posits that sleep results from interactions between Process S (sleep-wake homeostasis) and Process C (circadian rhythm; Borbély, 1982). In 2001, the “flip-flop” model of sleep-wake regulation emerged, suggesting mutual inhibition and promotion between sleep and wakefulness states (Saper et al., 2001). In this model, adenosine released by basal forebrain neurons accumulates during wakefulness and diminishes during sleep, serving as a critical molecular regulator of sleep homeostasis (Peng et al., 2020). As a universally observed biological phenomenon, sleep is typically defined as prolonged periods of inactivity with reduced environmental responsiveness requiring compensation following deprivation (Buysse, 2014). This appears highly conserved across diverse organisms, with sleep-like behaviors observed even in simple life forms such as jellyfish and nematodes (Nath et al., 2017; Raizen et al., 2008). The precise mechanisms underlying sleep in humans and mammals remain incompletely understood.

*Drosophila melanogaster* offers irreplaceable advantages as a model organism for sleep research. Approximately 60% of *Drosophila* genes show homology with mammalian counterparts (Singh et al., 2024), and fly sleep regulation by both circadian and homeostatic processes aligns closely with the mammalian two-process model (Huber et al., 2004; Abhilash and Shafer, 2024). While many studies have shown that sleep deprivation in fruit flies can induce sleep rebound (Dubowy et al., 2016; Ko et al., 2023a; Huang et al., 2024), it is important to note that this response is not universally observed. The occurrence of sleep rebound can depend on the specific neural circuits driving wakefulness, rather than merely the duration of the sleep deprivation (Seidner et al., 2015). The molecular mechanisms governing sleep (such as GABA signaling pathways and circadian-related genes) demonstrate evolutionary conservation (Chaturvedi et al., 2022; Konopka and Benzer, 1971). Optogenetic and chemogenetic approaches enable precise manipulation of specific neurons (e.g., dFB neurons), facilitating analysis of neural mechanisms underlying sleep homeostasis (Jones et al., 2025; Ni et al., 2019). Furthermore, *Drosophila* benefits from sophisticated genetic tools and gene editing technologies (including gene knockout, RNA interference, and tissue-specific expression systems), enabling efficient screening and validation of sleep-related genes and neural circuits (Shafer and Keene, 2021). Fly sleep behavior can be continuously and precisely quantified through automated systems (such as infrared activity monitoring and machine vision), measuring parameters including sleep duration, fragmentation degree, and arousal threshold (Xu et al., 2021; Churgin et al., 2019). Rapid reproduction and short lifespan (approximately 60–80 days) facilitate long-term or large-scale sleep studies (Dilley et al., 2018).

Both neural circuits and gene networks participate in *Drosophila* sleep regulation. At the neural circuit level, the dorsal fan-shaped body (dFB) interacts with the spiral neurons in the central complex by releasing inhibitory neurotransmitters, integrating sensory information, and regulating sleep pressure (Donlea et al., 2018). Circadian neurons (DN1p, DN3) modulate sleep–wake cycles (Lamaze et al., 2018; Guo et al., 2016; Jiang et al., 2025), while R5 ellipsoid neurons regulate dopaminergic neurons through astrocytic signaling to convey sleep requirements (Ho et al., 2022). At the genetic level, the Shaker potassium channel gene modulates temperature-adaptive sleep via GABAergic neurons (Kim et al., 2020); the *Fruitless* gene controls sex-specific interactions between sleep and sexual behavior (Chen et al., 2017); while genes such as *GAT* and *MPP6* participate in sleep homeostasis maintenance (Chaturvedi et al., 2022; Khoury et al., 2021). A recent study suggests that glial cells—particularly oligodendrocyte precursor cells (OPCs) and astrocyte-like AL cells—actively sense metabolic states and dynamically regulate sleep behavior through calcium signaling (Flores-Valle et al., 2025). This finding implies that these cells may also directly contribute to sleep regulation in *Drosophila*. These mechanisms collectively confer environmental adaptability, developmental regulation, and integration of sleep with memory. Research on *Drosophila* sleep using genetic and neuroscientific approaches provides crucial insights for understanding mammalian sleep mechanisms.

Large-scale connectomics studies in *Drosophila* have revealed that sleep-related neurons are not discrete centers but are embedded within widely distributed networks supporting sensorimotor integration (Schlegel et al., 2024; Dorkenwald et al., 2024). Ascending and descending neurons form bidirectional pathways linking sensory processing and sleep–wake states (Stürner et al., 2025), providing circuit-level explanations for sleep phenotypes and shifting the focus from isolated “sleep centers” to dynamic whole-brain network organization.

In parallel, molecular and genetic mechanisms within these networks are essential for regulating sleep. This review highlights key neural structures and sleep-regulatory genes, categorizes their roles, and summarizes insights from sleep mutation studies. Together, these findings offer an integrative perspective on how molecular, cellular, and circuit-level mechanisms interact to shape sleep behavior in *Drosophila*.

## 2 Neural structures regulating sleep in *Drosophila*

**Figure 1:**
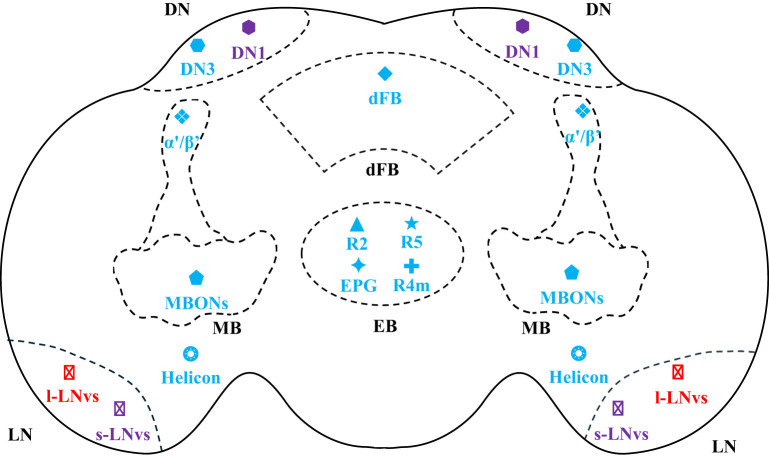
Distribution of the main neurons involved in sleep regulation in the *Drosophila* brain. Black fonts and their corresponding dashed lines represent anatomical locations in the *Drosophila* brain, blue fonts represent neurons that promote sleep, red fonts represent neurons that promote wakefulness, and purple fonts represent neurons that can promote both sleep and wakefulness.

*Drosophila* sleep is regulated by multiple brain regions working in concert, including the Fan-shaped Body (FB), Ellipsoid Body (EB), Mushroom Body (MB), and protocerebral circadian neurons (such as DN and LN neurons). These brain regions control sleep through mechanisms including electrical activity switching, neurotransmitter/neuromodulator signaling, synaptic plasticity, and environmental sensing, integrating sleep pressure, circadian rhythms, and memory requirements to achieve sleep regulation. Figure 1 shows the distribution of the main neurons involved in sleep regulation in the *Drosophila* brain.

### 2.1 Dorsal fan-shaped body neurons

The dFB plays a crucial role in sleep regulation in *Drosophila*, where its neurons control sleep by switching between different electrical activity states, such as active vs. resting configurations. Dopamine plays a crucial role in modulating these transitions by altering potassium channel activity, facilitating the switching between wakefulness and sleep (Pimentel et al., 2016). dFB neurons also release the inhibitory transmitter allatostatin-A onto AstA-R1-expressing helicon cells of the central complex, suppressing visually guided movement and consolidating sleep (Donlea et al., 2018). However, recent findings have challenged the role of AstA in dFB-mediated sleep regulation. De et al. (2023) show that 23E10+ dFB-projecting neurons do not express AstA RNA or protein, suggesting that AstA may not play a significant role in dFB-based sleep regulation, as previously thought.

Accumulating sleep pressure elevates mitochondrial ROS, which oxidize the K_V_β subunit Hyperkinetic, enhancing A-type currents and spike frequency in dFB neurons through altered NADPH/NADP^+^ ratios, thus driving sleep (Kempf et al., 2019). Upstream excitatory glutamatergic inputs from circadian neurons and inhibitory dopaminergic arousal signals converge on the dFB to coordinate sleep-wake balance (Ni et al., 2019). Developmental transcriptomic changes within dFB neurons sculpt age-dependent sleep patterns (Gong et al., 2022), while 5-HT2b receptor expression in these cells is required for adult sleep homeostasis. Recent single-cell analyses further reveal dFB heterogeneity: cholinergic and glutamatergic sub-populations preferentially regulate sleep homeostasis and memory consolidation, respectively, with activation thresholds higher than other sleep-promoting neurons like VNC-SP cells (Jones et al., 2025).

Recent research on *Drosophila* sleep has sparked debate regarding the role of the dFB. The traditional view suggests that the dFB is a key brain region involved in sleep homeostasis, primarily based on studies using genetic tools such as the 23E10-GAL4 driver. However, new evidence suggests that these drivers lack specificity and are also expressed in peripheral nervous system components, such as leg ppk neurons and cholinergic neurons in the ventral nerve cord (Jones et al., 2023; De et al., 2023; Satterfield et al., 2022). This raises the possibility that the phenotypes previously attributed to the dFB may, in part, be driven by peripheral neurons (De et al., 2023; Satterfield et al., 2022). The Joiner lab has shown that activating ppk neurons alone can induce sleep homeostatic responses, while blocking their activity eliminates phenotypes traditionally ascribed to central brain regions (Satterfield et al., 2022). This has led the Joiner lab to propose that the key regulators of sleep homeostasis may actually lie within ventral brain regions (SEZ/AMMC), which integrate signals from peripheral neurons, including ppk neurons (Satterfield et al., 2022).

The Dissel lab, using a dFB-specific Split-GAL4 tool, demonstrated that intense activation of dFB neurons promotes sleep, whereas inhibition disrupts sleep homeostasis (Jones et al., 2025). Their findings reveal that the dFB is primarily composed of cholinergic and glutamatergic neurons, with the cholinergic subset playing a critical role (Jones et al., 2025). These conflicting results underscore the importance of genetic tool specificity. Many commonly used GAL4 drivers co-express background neurons such as those in the VNC-SP, necessitating caution in attributing phenotypes (Jones et al., 2023, 2025). A growing consensus in the field emphasizes the need for more specific genetic tools and standardized protocols to better delineate the roles of different neurons in sleep regulation (De et al., 2023).

Current models are shifting from a view of sleep regulation centered on a single “hub” (e.g., the dFB) to one that involves a distributed network of neurons. Peripheral sensory neurons, like ppk, act as sensors for wakefulness, while ventral brain regions integrate these signals, and higher brain regions such as the dFB set thresholds for sleep behavior (Satterfield et al., 2022; Jones et al., 2025). Future efforts should leverage connectomic data to map a comprehensive sleep regulation pathway from the periphery to the central brain, with the aim of resolving these ongoing controversies.

### 2.2 Ellipsoid body R2 and R5, EPG neurons

*Drosophila* ellipsoid-body (EB) neurons contribute to sleep homeostasis, sleep architecture and dopaminergic signaling. R5 EB neurons act as sleep drivers; during deprivation their firing shifts from tonic to burst mode, encoding sleep pressure through synaptic plasticity that includes increased NMDA-receptor expression and elevated calcium signaling (Liu et al., 2016). These neurons excite EPG cells via cholinergic transmission, and the strength of this connection rises with accumulated sleep need (Ho et al., 2022). Other EB sub-populations shape sleep structure: R2 and R4m neurons interact with dopaminergic systems to modulate memory formation in delay and trace conditioning through D1- and D2-like receptors, respectively (Grover et al., 2022), whereas R2 neurons that target helicon cells promote sleep by suppressing visually guided locomotion (Donlea et al., 2018). EB circuits also stabilize sleep continuity by inhibiting tubercular-bulbar (TuBu) neurons in the anterior optic tubercle (Lamaze et al., 2018). Serotonergic modulation of EB circuits increases sleep fragmentation without altering total sleep time, thereby impairing learning (Liu et al., 2019). Finally, during low-temperature diapause, calcium signaling and the presynaptic marker Bruchpilot are up-regulated in R5 neurons, reinforcing their role in integrating sleep pressure (Meyerhof et al., 2024).

### 2.3 Mushroom body α′/β′ neurons

*Drosophila* mushroom bodies (MBs) contribute to both memory consolidation and sleep regulation through distinct circuits and molecular pathways. When food is abundant, anterior–posterior α′/β′ neurons promote sleep-dependent memory consolidation by increasing sleep; under starvation, medial α′/β′ neurons support sleep-independent memory, demonstrating environment-dependent switching between sleep and memory modes (Chouhan et al., 2021). Developmental or functional perturbation of MB neurons—e.g., mutation of the *insomniac* gene—disrupts sleep architecture (Li et al., 2021), and sleep loss alters synaptic plasticity within the MBs, including elevated presynaptic Bruchpilot (BRP) expression, indicating that sleep maintains MB circuit homeostasis (Weiss and Donlea, 2021). MB output neurons (MBONs) direct distinct memory-guided behaviors by decoding memory valence (reward vs. punishment), underscoring the MB's capacity to integrate sleep and memory processes (Ichinose et al., 2021). Calcium signaling via Neurocalcin and molecular rhythms controlled by the NF1–cAMP/PKA pathway in MB neurons regulate night-time sleep and circadian arousal (Chen et al., 2019; Almeida et al., 2021), whereas rhythmic switching of transcriptional regulators (CREB/CRTC and Bx) and RNA-processing factors (*Polr1F* and *Regnase-1*) reveals epigenetic and translational mechanisms that couple sleep to memory consolidation (Hirano et al., 2016; Li Y. et al., 2024).

### 2.4 Protocerebral dorsal DN neurons

DN neurons integrate environmental information with molecular clock outputs through multisynaptic circuits, neuropeptide signaling, and transcriptional regulation to control sleep-wake rhythms. DN1 neurons (DN1ps) can be divided into morphologically and functionally distinct subgroups: one subset promotes wakefulness by inhibiting TuBu neurons in the anterior optic tubercle (AOTU), which connect to ring neurons in the central complex's EB, regulating sleep-wake timing and arousal states (Lamaze et al., 2018; Guo et al., 2018); another subset promotes sleep by releasing glutamate to directly inhibit key pacemaker neurons (Lamaze et al., 2018). Additionally, DN1 neurons regulate evening activity through the Allatostatin-C (AstC) neuropeptide signaling pathway, with AstC oscillating in DN1s and inhibiting LNd neurons via AstC-R2 receptors, influencing photoperiod-dependent behavioral rhythms (Díaz et al., 2019). DN1 neuronal activity also exhibits gender and environmental temperature dependence, with higher DN1 activity in male flies correlating with increased daytime sleep, while high temperatures enhance DN1 activity to promote sleep (Kim et al., 2020). Further research reveals that DN1 neurons form a positive feedback loop with anteriorly projecting DN3 neurons (APDN3s), which maintain sleep stability by activating non-rhythmic “claw neurons” (CLs) that release acetylcholine acting on the mushroom body γ lobe (Sun et al., 2022). DN3 neurons themselves display high heterogeneity, with single-cell transcriptome analysis revealing 12 subgroups, some of which promote sleep through the TrissinR receptor pathway (Ma et al., 2025).

### 2.5 Protocerebral lateral neurons (LN)

*Drosophila* LN neurons divide into large ventrolateral neurons (l-LNvs) and small ventrolateral neurons (s-LNvs). Research shows that large ventrolateral LN neurons (lLNvs) influence sleep through rhythmic regulation of GABAergic signaling: E3 ligase Fbxl4, dependent on clock gene *CLOCK* transcription, promotes ubiquitination and degradation of GABA_A_ receptors, reducing lLNvs' sensitivity to GABA, enhancing their excitability and promoting wakefulness (Li et al., 2017). Additionally, dynamic reconstruction of LN neuron networks adapts to changes in light environment: morning-active s-LNv neurons form cascade or parallel circuits with DN1p or LNd neurons through PDF neuropeptide, with light signals reshaping coupling relationships between these oscillators—s-LNvs preferentially couple with LNd in light while selecting DN1p in darkness, adjusting behavioral output timing (Chatterjee et al., 2018). Further research reveals that evening oscillators (E cells, including LNd and PDF-negative sLNv) provide excitatory feedback to s-LNv (M cells) through acetylcholine and glutamate, with this synaptic connection's circadian plasticity (such as enhanced excitatory input in the evening) crucial for maintaining robust rhythmic output (Duhart et al., 2020b). Astrocytes also participate in regulating LN neurons' sleep function, with their GABA transporter (GAT) modulating sleep quality and duration by reducing GABAergic tone around lLNvs, inhibiting GABA_A_ receptor RDL activation (Chaturvedi et al., 2022). Recent research discovered that two LN neurons expressing ITP play important roles in morning activity, with light-dependent upregulation of their dopamine receptor Dop1R1 enhancing morning cAMP responses to dopamine, promoting wakefulness—this mechanism reveals the molecular basis for light and dopamine's synergistic regulation of morning activity (Le et al., 2024).

### 2.6 Dopaminergic neurons across multiple brain regions

*Drosophila*'s dopamine (DA) system displays complex region-specific functions in sleep regulation. Research shows that dopamine receptor DopR1 (DopR) in the MB regulates daytime sleep, with DopR expression in specific MB neurons promoting sleep by inhibiting Kenyon cells (KCs) in γ5 and β′2 regions and mushroom body output neurons (MBONs; Jiang et al., 2016; Driscoll et al., 2021). Conversely, dFB neurons in the central complex (CX) mediate nighttime sleep regulation through dopaminergic signaling: DA activates potassium channels (like Sandman) via Dop1R2 receptors, switching dFB neurons from excited (ON) to resting (OFF) states, thereby promoting sleep (Pimentel et al., 2016; Dissel et al., 2022). Additionally, dopaminergic signaling in clock neurons exhibits dual effects: although DA excites large ventrolateral neurons (l-LNvs) through Dop1R1 receptors, its arousal effect is inhibited, while Dop1R2 signaling in small ventrolateral neurons (s-LNvs) promotes nighttime sleep (Fernandez-Chiappe et al., 2020). Dopaminergic neurons in the protocerebral bridge (PB; T1 DA) enhance wakefulness by inhibiting sleep-promoting neurons via Dop2R receptors (Tomita et al., 2021). Nutritional status further regulates dopaminergic circuits; for example, hunger switches MB α′/β′ neurons from sleep-dependent to sleep-independent memory by inhibiting neuropeptide F (NPF) signaling (Chouhan et al., 2021), while DA-PB neurons preferentially inhibit sleep to promote courtship behavior when nutrition is abundant (Duhart et al., 2020a). Furthermore, sleep-dependent memory consolidation involves reactivation of dopaminergic neurons, with fan-shaped body neurons enhancing long-term memory by triggering replay in DA neurons (Dag et al., 2019). These studies indicate that dopamine dynamically coordinates sleep-wake balance through receptor subtypes and neural circuits in different brain regions to adapt to environmental demands.

### 2.7 Octopaminergic neurons across multiple brain regions

In *Drosophila*, octopamine functions as a neuromodulator similar to mammalian norepinephrine, playing an important role in sleep regulation. During the larval period, octopamine serves as the primary arousal regulator, affecting developmental sleep by promoting neural stem cell proliferation, while dopamine does not participate in regulation at this stage (Szuperak et al., 2018). In adult flies, octopaminergic neurons (such as MS1 neurons) mediate sex-driven sleep inhibition by forming male-specific synaptic connections with non-sex-specific *FRUITLESS (FRU)* neurons (Machado et al., 2017). Further research reveals that nutritional status regulates sleep-courtship balance through octopaminergic neurons, with dopaminergic neurons projecting to the protocerebral bridge (DA-PB) acting as downstream targets of P1 neurons to promote wakefulness when food is abundant (Duhart et al., 2020a). Additionally, octopamine receptor Octβ2R in the ellipsoid body is regulated by epigenetic factor Stuxnet-Polycomb cascade, affecting sleep homeostasis (Zhao et al., 2021). In mushroom body circuits, octopaminergic neurons from the subesophageal zone inhibit GABAergic MBON neurons by activating anterior medial protocerebral dopaminergic neurons (PAM), maintaining the wakeful state needed for prolonged flight (Manjila et al., 2019). dFB sleep homeostatic neurons inhibit octopaminergic arousal neurons through GABAergic signaling, while receiving bidirectional regulation from circadian neurons and dopaminergic neurons (Ni et al., 2019). At the molecular level, octopamine promotes sleep through different receptor subtypes (such as nAChRα2/β2) in octopaminergic neurons, while promoting external stimulus-induced arousal in dopaminergic neurons through nAChRα3 (Dai et al., 2021). Notably, optic lobe glial cells show significantly higher sensitivity to Ca^2+^ responses to low concentrations of octopamine than neurons, suggesting glial cells may regulate synaptic plasticity and visual processing through octopamine signaling, thereby influencing sleep (Cerne et al., 2025).

### 2.8 Glutamatergic neurons across multiple brain regions

In *Drosophila*, glutamate, a neurotransmitter traditionally associated with arousal, plays a crucial regulatory role across multiple brain regions. The activity of glutamatergic neurons in the adult *Drosophila* brain significantly promotes wakefulness, with increased neuronal activity extending wake periods in the circadian cycle. Conversely, when activity is suppressed or neurotransmission is blocked, the duration of nocturnal wakefulness is reduced (Zimmerman et al., 2017). Dorsal clock neurons (DN1s) directly inhibit key pacemaker neurons by releasing glutamate, forming a feedback regulatory loop that promotes both midday naps and nocturnal sleep (Guo et al., 2016). In aging *Drosophila*, a decline in the function of metabotropic glutamate receptors (*mGluR*) is closely associated with sleep disturbances and memory decline, while overexpression of *mGluR* can improve sleep (Hou et al., 2023). Additionally, subsets of glutamatergic and cholinergic neurons in the dFB specifically regulate sleep homeostasis and memory consolidation (Jones et al., 2025).

The role of glutamate in sleep regulation extends beyond interactions between neurons. Amyloid precursor protein (Appl) in glial cells influences sleep by regulating genes associated with glutamate recycling, such as glutamine synthetase and the glutamate transporter dEaat1 (Farca Luna et al., 2017). Furthermore, inhibiting the electron transport chain in glutamatergic neurons can extend lifespan and increase sleep, suggesting a close link between the metabolic state of these neurons and sleep regulation (Landis et al., 2023). During aging, the loss of DmGluRA exacerbates sleep fragmentation and shortens lifespan (Ly and Naidoo, 2019), while the RNA editing gene Adar regulates sleep pressure by suppressing glutamatergic synaptic plasticity (Robinson et al., 2016). Metabolic studies further indicate that sleep deprivation disrupts glutamate metabolic pathways, reinforcing glutamate's crucial role in maintaining the sleep-wake balance (Malik et al., 2024).

However, despite the traditional view of glutamate as an arousal-promoting neurotransmitter, recent research has revealed that glutamate, through activation of the glutamate-gated chloride channel (GluClα), inhibits the activity of two pairs of neurons in the ventral nerve cord (VNC), thereby promoting nocturnal sleep (Fan et al., 2025). This finding challenges the conventional understanding of glutamate's role in arousal and uncovers a novel role for VNC neurons in sleep regulation. Taken together, glutamate regulates sleep dynamically across different regions of the *Drosophila* brain through multiple mechanisms, including ionotropic and metabotropic receptors, glial-neuronal interactions, and metabolic regulation.

### 2.9 Cholinergic neurons across multiple brain regions

Different acetylcholine receptors (AChRs) and neuronal subgroups play specific roles in sleep and wakefulness. Among *Drosophila*'s 13 AChRs, nAChRα3 promotes exogenous stimulus-induced arousal through dopaminergic neurons, while nAChRα2 and β2 promote sleep through octopaminergic neurons, revealing paradoxical mechanisms by which a single neurotransmitter regulates sleep and wakefulness through different receptors and neuron types (Dai et al., 2021). Cholinergic neurons in the ventral nerve cord (VNC-SP) regulate baseline sleep through the 23E10-GAL4 driver but do not participate in sleep homeostasis regulation, suggesting neuronal heterogeneity in sleep control (Jones et al., 2023). Subsequent research further shows that the dFB, as a key sleep regulation region, contains neurons with neurochemical heterogeneity: most dFB neurons co-express glutamate and acetylcholine, few express only one, and cholinergic dFB neurons play crucial roles in sleep homeostasis and memory consolidation (Jones et al., 2025). Sleep need transmits sleep drive by enhancing cholinergic synaptic connections between R5 neurons and EPG neurons, with EPG neurons showing increased spontaneous firing after sleep deprivation, demonstrating dynamic regulation of cholinergic signaling in sleep homeostasis (Ho et al., 2022). Additionally, alcohol-induced long-term sleep deficits correlate with cholinergic neuron activity inhibition, particularly functional impairment of mushroom body cholinergic neurons, further emphasizing the cholinergic system's importance in sleep disorders (Chvilicek et al., 2025).

## 3 Genes involved in *Drosophila* sleep regulation

With continuous advances in molecular biology techniques, an increasing number of genes participating in *Drosophila* sleep regulation have been identified. These genes directly or indirectly modulate sleep in *Drosophila*. In this review, we adopt the classification method of Afonso D. J. et al. (2015) to categorize these sleep-regulatory genes according to their biological functions, and have compiled the sleep parameters of gene mutants reported in the literature in Supplementary material Table 1.

### 3.1 Neurotransmitter-related genes

The serotonergic (5-hydroxytryptamine, 5-HT) system regulates sleep through diverse receptor subtypes. The 5-HT1A receptor promotes baseline sleep, with mutations resulting in shortened and fragmented sleep periods; expression of this receptor in the MB restores sleep phenotypes, indicating region-specific serotonergic sleep regulation (Yuan et al., 2006). 5-HT2B receptors, expressed in a subset of dFB neurons, maintain sleep homeostasis, with their absence attenuating post-sleep deprivation rebound (Qian et al., 2017). Both 5-HT1A and 5-HT2B receptors influence sleep architecture by modulating neural activity in the MB and central complex, respectively (Bonanno et al., 2024). Interactions between 5-HT and other neurotransmitters also contribute to sleep regulation; for instance, GABA/5-HTP mixtures extend nocturnal sleep duration in *Drosophila* by upregulating GABA_B_ receptor and 5-HT1A receptor expression (Hong et al., 2016). Furthermore, gut microbiota dysbiosis (such as reduced *Lactobacillus plantarum* populations) or high palmitic acid diets disrupt sleep-wake homeostasis by inhibiting *5-HT1A* expression through inflammatory mediators (including *Upd3*; Huang et al., 2024). Adipose tissue-derived exosomes containing circ_sxc indirectly regulate 5-HT1B and other receptor expression by sequestering *miR-87-3p*, influencing age-related sleep disorders (Li Q. et al., 2024).

The dopamine transporter (DAT) modulates sleep behavior and arousal thresholds in *Drosophila*. Studies have found that *DAT* gene mutations (such as the *fumin* mutant) lead to a significant reduction in sleep duration, characterized by an inability to maintain sleep states and a lowered arousal threshold, although sleep homeostasis (such as the rebound after sleep deprivation) may remain normal or be enhanced (Bi et al., 2018; Kume et al., 2005). This phenotype is associated with hyperactivation of dopaminergic signaling, as DAT deficiency inhibits the reuptake of dopamine in the synaptic cleft, thereby continuously activating downstream pathways (Kume et al., 2005). Further research indicates that dopamine regulates arousal in the dFB through the D1 receptor (DA1); the short-sleep phenotype of *DAT* mutants can be fully rescued by *DA1* gene mutations, and the specific expression of *DA1* in the dFB restores the short-sleep phenotype (Ueno et al., 2012). Additionally, DAT function is regulated by molecular chaperones (such as noribogaine and HSP70 inhibitors), which can rescue sleep abnormalities due to DAT folding defects, for example, restoring DAT synaptic localization and sleep duration in fumin mutants (Sucic et al., 2016; Asjad et al., 2017). Wolbachia infection, an endosymbiotic bacterium, also increases total sleep time in *Drosophila* by upregulating the expression of dopaminergic synthesis genes *Pale* and *Ddc*, but reduces sleep quality (manifested as fragmented sleep and decreased arousal threshold), suggesting that host-microbe interactions may indirectly affect DAT function through dopaminergic pathways (Bi et al., 2018). DAT is also involved in the regulation of sleep by amphetamines (AMPH); AMPH increases dopamine release in a DAT-dependent manner, leading to sleep fragmentation and hyperactivity, whereas *DAT* mutant responses to AMPH result in activity inhibition and sleep recovery (Fagan et al., 2021; Karam et al., 2022). Moreover, high-calorie diets exacerbate age-related sleep disorders and shortened lifespan in *DAT* mutants, indicating that dopaminergic signaling and metabolic regulation jointly influence sleep (Yamazaki et al., 2012).

Dopamine receptor-related genes in *Drosophila* exhibit complex and diverse functions in sleep regulation. Different receptor subtypes (such as Dop1R1 and Dop1R2) bidirectionally modulate sleep-wake states. For instance, Dop1R2 in dFB neurons mediates dopaminergic signaling to promote sleep by regulating potassium channels (like Sandman) that switch neuronal states (Pimentel et al., 2016). Conversely, Dop1R1 increases sleep in central complex neurons by suppressing repeated startle-induced arousal (Lebestky et al., 2009). Additionally, Dop1R1 expression in ion transport peptide-positive (ITP+) circadian neurons is light-regulated, with enhanced cyclic adenosine monophosphate (cAMP) signaling responsiveness to dopamine in the morning, facilitating morning wakefulness (Le et al., 2024). Interactions between dopamine and other neurotransmitters also regulate sleep; for example, 14-3-3ε influences sleep by controlling Dop1R1 and octopamine receptor (Oamb) expression (Wei et al., 2021), while the Wolfram syndrome gene *wfs1* affects sleep by modulating calcium homeostasis and excitability in Dop2R neurons (Hao et al., 2023). Dopaminergic signaling also interacts with environmental factors, as larval malnutrition upregulates Dop1R2 expression through epigenetic modifications, resulting in increased activity and reduced sleep in adults (Zúñiga-Hernández et al., 2023). Dopamine receptors function differently across neural circuits; Dop1R1 in mushroom body Kenyon cells regulates startle-induced locomotion (Sun et al., 2018), whereas Dop2R in protocerebral bridge (PB) neurons reduces sleep by inhibiting sleep-promoting neuronal activity (Tomita et al., 2021).

Different acetylcholine receptors (AChRs) distinctly regulate sleep and wakefulness by acting on specific neuronal populations. The nicotinic acetylcholine receptor α3 (nAChRα3) promotes exogenous stimulus-induced arousal through dopaminergic neurons, while nAChRα2 and nAChRβ2 promote sleep through octopaminergic neurons, resolving the apparent paradox of acetylcholine's dual role in both sleep promotion and arousal (Dai et al., 2021). Forward genetic screening identified the *redeye* (*rye*) gene, which encodes a nicotinic acetylcholine receptor α subunit. RYE protein levels oscillate throughout light-dark cycles, peaking during daily sleep periods. Its expression is regulated by sleep homeostasis, with sleep deprivation or short-sleeping mutants triggering RYE upregulation, indicating that RYE promotes sleep in response to sleep pressure (Shi et al., 2014).

γ-Aminobutyric acid (GABA) and its associated genes regulate sleep through neuron-glia interactions, metabolic balance, and neural circuit modulation. GABA transporters (GAT) in astrocytes influence sleep by regulating GABAergic neurotransmission, as evidenced by *gat* mutant (*gat33-1*) flies exhibiting increased sleep duration and shortened sleep latency. This phenotype depends on the expression of GABA_A_ receptor RDL and its regulatory factors wide *awake (wake)* and *Drosophila neuroligin 4 (dnlg4)* in arousal-promoting l-LNvs (Chaturvedi et al., 2022). Deficiency in the GABA-degrading enzyme GABA transaminase (GABA_T_) increases sleep, while GABA_T_ dysfunction in glial cells (such as elevated GABA_T_ levels in sleepless mutants) promotes wakefulness by reducing brain GABA levels, highlighting the critical role of neuron-glia interactions in sleep regulation (Chen et al., 2015; Maguire et al., 2015). GABA_T_'s metabolic function operates independently of sleep regulation; it generates glutamate through GABA degradation, influencing the tricarboxylic acid cycle and energy metabolism without directly mediating sleep phenotypes (Maguire et al., 2015). At the neural circuit level, dorsal paired medial (DPM) neurons promote sleep by releasing GABA to inhibit α′/β′ neurons in the MB, potentially coordinating sleep with memory consolidation through this mechanism (Haynes et al., 2015).

In *Drosophila*, octopamine and its associated genes influence sleep through multilayered neural circuits and molecular pathways. Octopamine modulates sleep-wake behavior by activating specific receptors, including octopamine receptor in mushroom bodies (OAMB), *Oct*β*1R, Oct*β*2R*, and *Oct*α*2R*. Octopaminergic neurons (ASM cells) promote wakefulness by acting on insulin-producing neurons in the PI through the OAMB receptor (cAMP-dependent subtype), a mechanism analogous to neuroendocrine regulation in the mammalian hypothalamus (Crocker et al., 2010). The Octα2R receptor, expressed throughout the central nervous system, regulates motor control and behavioral modulation, as its functional loss results in decreased locomotor activity and increased grooming behavior (Nakagawa et al., 2022). Epigenetic mechanisms also participate in octopamine receptor expression; the Stuxnet-Polycomb-Octβ2R cascade positively regulates *Oct*β*2R* expression by inhibiting the Polycomb complex, thereby influencing sleep homeostasis (Zhao et al., 2021). Recent studies utilizing gene editing techniques have mapped the subcellular localization of octopamine receptors (such as OAMB), revealing their enrichment at neural terminals within the mushroom bodies and visual system, suggesting potential involvement in synaptic transmission within sleep-related neural circuits (Bonanno et al., 2024).

### 3.2 Cell signaling pathway-related genes

*Drosophila* insulin/PI3K/AKT pathway-related genes indirectly regulate sleep by modulating metabolism, neuroplasticity, and circadian function. Mutations in *Drosophila* insulin-like peptides (*DILPs*) and their receptor (*DInR*) reduce sleep duration, while neuronal overexpression of *DILPs* increases sleep time, indicating positive regulation of sleep by insulin signaling (Cong et al., 2015; Yamaguchi et al., 2022). *DILP2* levels decrease under starvation conditions, correlating with sleep suppression and further confirming insulin signaling's role in metabolic-sleep interactions (Brown et al., 2020). Insulin signaling modulates sleep through dorsal neurons 1 anterior (DN1a) and pars intercerebralis (PI) neurons, with inhibition of insulin receptor (InR) in either DN1a or PI reducing sleep (Yamaguchi et al., 2022). Insulin signaling also regulates sleep via the target of rapamycin (TOR) pathway; TOR signaling and autophagy-related genes (such as *Atg5* and *Atg7*) exhibit circadian expression in wild-type *Drosophila* brains, which is abolished in the circadian mutant *per01*, suggesting coordinated sleep regulation by insulin/TOR pathways and the circadian clock (Kijak and Pyza, 2017). Sleep deprivation alters expression of clock genes and DILPs while disrupting glucose, triglyceride, and glycogen metabolism, indicating insulin signaling mediates the negative effects of sleep deprivation on metabolism and circadian rhythms (Rodrigues et al., 2023). Reducing insulin/IIS/TOR network activity ameliorates age-related sleep fragmentation, with nighttime sleep continuity and consolidation dependent on S6K and dopaminergic signaling (Metaxakis et al., 2014). Insulin signaling also influences neuronal stress responses through the Akt-GSK3β pathway, with photoperiod alterations reducing Akt phosphorylation levels and consequently affecting sleep stability (Moore et al., 2021). Gut microbiota (such as *Bifidobacterium adolescentis*) may promote sleep by upregulating insulin receptor (*InR*) gene expression, further extending insulin signaling's multisystemic role in sleep regulation (Ko et al., 2023b).

The salt-inducible kinase 3 (*Sik3*) gene exhibits remarkable evolutionary conservation across species. The *Sleepy* mutation (caused by exon-skipping in the *Sik3* gene) and *Sik3-SA* (serine-to-alanine at a protein kinase A phosphorylation site) in mice induce increased sleep (Funato et al., 2016; Honda et al., 2018); consistently, the *Drosophila Sik3* homolog similarly participates in sleep regulation (Funato et al., 2016). Furthermore, research has shown that neuronal overexpression of a *Sik3* variant with a critical phosphorylation-site mutation, *Sik3-SA*, increases sleep duration in flies under both light-dark (LD) and constant darkness (DD) conditions (Kobayashi et al., 2023). When *Sik3-SA* is specifically overexpressed in pigment-dispersing factor (PDF) neurons—central pacemaker neurons controlling circadian rhythms—subjective daytime sleep increases while circadian amplitude decreases; conversely, in flies with pan-neuronal *Sik3-SA* overexpression, selective inhibition of *Sik3-SA* expression in PDF neurons reverses the subjective daytime sleep increase (Kobayashi et al., 2023). These findings demonstrate that *Sik3* modulates circadian-related sleep behaviors through PDF neurons, likely via phosphorylation-dependent signaling pathways.

Calcium signaling genes in *Drosophila*, particularly calcineurin (CN) and its regulatory pathways, play integral roles in sleep regulation. Pan-neuronal RNA interference-mediated knockdown of CN reduces sleep in flies, while overexpression of the constitutively active form of the CN catalytic subunit (CnA) increases sleep duration, demonstrating a positive correlation between CN activity levels and sleep time (Tomita et al., 2011). These findings align with Nakai et al. (2011), who observed that deletion of CN catalytic subunit *CanA-14F* and regulatory subunit CanB significantly diminishes sleep, with aberrant CN activity—whether elevated or reduced—disrupting sleep homeostasis. Furthermore, CN function extends to memory regulation, as its knockdown impairs aversive olfactory memory retention in flies, suggesting that CN may coordinate sleep-memory interactions through calcium-dependent signaling pathways (Tomita et al., 2011). Additionally, the Rho-GTPase activating protein Crossveinless-c (Cv-c) translates sleep pressure into neuronal activity changes by modulating the electrical excitability of dFB neurons. *cv-c* mutants exhibit reduced sleep, attenuated sleep rebound, and memory deficits—phenotypes reminiscent of sleep deprivation (Donlea et al., 2014).

The *Drosophila foraging (for)* gene, encoding cyclic guanosine monophosphate (cGMP)-dependent protein kinase (PKG), orchestrates multiple behavioral phenotypes including sleep regulation, environmental stress tolerance, and age-related functional decline. For influences functional aging in *Drosophila* through the nitric oxide (NO)/cGMP/PKG signaling pathway. Alleles conferring high PKG activity enhance hypoxic stress tolerance while simultaneously reducing lifespan and age-dependent locomotor capacity, suggesting a pivotal role for PKG signaling in healthy aging and sleep-wake regulation (Kelly and Dawson-Scully, 2019). Downstream pathways of *for* modulate behaviors, including sleep, through tissue-specific transcriptomic dynamics. Food deprivation induces for allele-dependent gene expression changes in larval tissues, with differentially expressed genes involved in energy metabolism and neuromodulatory pathways potentially linked to sleep-wake cycle regulation (Sokolowski et al., 2023). G protein-coupled receptor kinase 2 (GPRK2), a putative PKG-interacting protein, regulates ethanol sensitivity and rapid tolerance in ellipsoid body neurons, while GPRK2 functional deficiency results in reduced and fragmented sleep, indicating that PKG-related pathways may modulate sleep homeostasis through G protein signaling (Kang et al., 2020). Additionally, neuropeptides such as PDF and short neuropeptide F (sNPF) regulate motor neuron activity through antagonistic electrophysiological effects: PDF promotes wakefulness by elevating cAMP levels, while sNPF inhibits neuronal activity through Gαo signaling-mediated cAMP reduction, suggesting PKG may participate in sleep-wake balance through interactions with these neuropeptide pathways (Vecsey et al., 2014).

Kinase and phosphatase regulatory genes play roles in sleep modulation. c-Jun N-terminal kinase [JNK, encoded by *Drosophila basket* (*bsk*)] regulates sleep and lifespan. Pan-neuronal knockdown of JNK in neurons results in reduced sleep and shortened lifespan, with particularly pronounced effects in mushroom body neurons, suggesting that JNK may regulate sleep through mechanisms independent of insulin signaling (Takahama et al., 2012). JNK is associated with synaptic pruning following neuronal injury; its functional deficiency attenuates the sleep-promoting effects after antennal injury, indicating that JNK might mediate sleep need through clearance of damaged neurons (Singh and Donlea, 2020). N-methyl-D-aspartate (NMDA) receptor-mediated Ca^2+^ influx (regulated by Mg^2+^ blockade) also influences circadian rhythms and sleep behavior. Mutants with defective Mg^2+^ blockade exhibit disrupted rest-activity rhythms, demonstrating that NMDA receptor Mg^2+^ blockade maintains sleep-wake balance by inhibiting the Ca^2+^/calmodulin (CaM)-activated phosphodiesterase 1c (PDE1c) pathway (Song et al., 2017). Overexpression of dual-specificity tyrosine phosphorylation-regulated kinase 1A [DYRK1A, encoded by *Drosophila minibrain (mnb)*] leads to decreased sleep and memory deficits. DYRK1A inhibitors (such as PST-001, DYR219, and DYR533) rescue sleep disturbances and neurodegenerative phenotypes caused by *Tau*, β*-amyloid*, or *mnb* overexpression, suggesting that DYRK1A may participate in sleep regulation through phosphorylation of substrates including Tau (Zhu et al., 2022a,b).

### 3.3 Neuropeptide signaling-related genes

*Drosophila* insulin-like peptides (DILPs) and their receptor (DInR) integrate nutritional status with sleep regulation through interactions between peripheral tissues and the brain. Most DILP mutants (such as *dilp2, dilp3, dilp5*) and DInR mutants exhibit reduced total sleep time, while upregulation of DILPs or DInR in the nervous system increases sleep duration (Cong et al., 2015). DILP2 levels decrease significantly during starvation, consistent with starvation-induced sleep suppression, indicating DILP system involvement in sleep regulation (Cong et al., 2015). DILP2-deficient flies demonstrate compensatory sleep rebound following starvation-induced sleep deprivation, suggesting DILP2 plays an essential role in recovery from sleep deprivation (Brown et al., 2020). Activation of insulin signaling in specific neurons (such as Drosulfakinin neurons) reduces sleep during fed states and attenuates starvation-induced sleep suppression, indicating that insulin signaling modulates metabolism and sleep behavior through neuronal circuits (Palermo et al., 2022). Adipose tissue-secreted cytokines (such as unpaired 2, *upd2*) also participate in sleep regulation; their downregulation inhibits sleep and enhances visual attention, potentially integrating into the brain through insulin-expressing cells (Ertekin et al., 2020). The Tribbles pseudokinase influences sleep by negatively regulating insulin signaling, with its overexpression reducing *DILP2* levels and increasing nighttime sleep duration (Popovic et al., 2023).

Diuretic hormone 31 (DH31) and diuretic hormone 44 (DH44) regulate *Drosophila* sleep behavior through multilayered neural circuits and molecular mechanisms, with functions contingent upon specific neuronal populations and developmental energy states (Yoon et al., 2025; Goda et al., 2016). DH31, secreted by dorsal clock neurons (DN1), inhibits nocturnal sleep and promotes wakefulness, particularly before dawn, through the pigment-dispersing factor receptor (PDFR) signaling pathway (Kunst et al., 2014). DH31 also modulates sleep via central complex neurons in the dorsal and ventral fan-shaped body (dFB and vFB) and ventral lateral clock neurons (LNvs); its secretion potentially influences dFB through autoregulatory feedback loops acting on DH31 receptors (Lyu et al., 2023). DH44 primarily regulates sleep homeostasis through interactions with insulin-producing cells and corticotropin-releasing factor (CRF)-homologous neurons (DH44 neurons). DH44 neurons mediate sleep suppression under starvation conditions (Oh and Suh, 2023), while under normal conditions, DH44 works synergistically with DH31 to influence sleep by modulating postsynaptic potentials and action potential timing precision in pars intercerebralis (PI) neurons (Chong et al., 2025). DH44 also participates in the developmental establishment of sleep-wake rhythmicity, with connections to DN1a clock neurons regulating the emergence of sleep rhythms during larval stages, subsequently affecting long-term memory formation (Poe et al., 2023, 2024). Notably, sleep in *Drosophila* larvae plays a crucial role in early neurodevelopment, including regulating neural stem cell proliferation (Szuperak et al., 2018). Moreover, advanced methods in larvae, such as the combined use of optogenetics and thermogenetics to artificially induce associative memory, provide powerful tools for dissecting the neurocircuitry underlying memory formation (Honda et al., 2016). Further studies using the advanced optogenetic and thermogenetic methods in *Drosophila* larvae will facilitate understanding of the development of the sleep-wake cycle and memory consolidation (Honda, 2022).

Pigment Dispersing Factor (PDF), a critical output signal from clock neurons, integrates light, temperature, and environmental information to regulate sleep and rhythmic behaviors through polysynaptic circuits (Lear et al., 2009; Shang et al., 2008). Secreted by ventral lateral neurons (LNvs), PDF signals through the PDF receptor (PDFR) to modulate dopaminergic neuron activity, promoting daytime wakefulness (Potdar and Sheeba, 2018). PDF neurons (particularly small LNvs, s-LNvs) establish temperature preference before dawn by activating dorsal neurons (DN2s), indirectly influencing sleep-wake rhythmicity (Tang et al., 2017). In warm environments, PDF signaling suppresses nocturnal activity, maintaining nighttime sleep stability (Iyengar et al., 2022). In Alzheimer's disease (AD) and Parkinson's disease (PD) models, abnormal axonal branching of PDF neurons or α-synuclein (α*-syn*) overexpression disrupts PDF signaling, resulting in sleep fragmentation and circadian rhythm disturbances (Song et al., 2016; Chen et al., 2025). PDF works synergistically with ion transport peptide (ITP) to regulate *Drosophila* nocturnal activity, with double knockout of PDF and ITP leading to hyperactivity and rhythm loss (Hermann-Luibl et al., 2014). Electrophysiological studies reveal that PDF depolarizes target neurons by increasing cyclic adenosine monophosphate (cAMP) levels, while other neuropeptides (such as short neuropeptide F, sNPF) exert opposing effects, indicating that PDF influences sleep-wake balance through modulation of neuronal excitability (Vecsey et al., 2014).

Appetite-related neuropeptide systems integrate metabolic demands with sleep regulation through multilayered neural circuits and molecular mechanisms. sNPF and its receptor promote sleep consolidation by inhibiting wake-promoting neurons, such as large ventral lateral clock neurons, and participate in sleep homeostasis regulation, a process involving cyclic adenosine monophosphate-protein kinase A-cAMP response element-binding protein (cAMP-PKA-CREB) signaling pathway activation (He et al., 2013; Shang et al., 2013). The neuropeptide F (NPF) system functions as a hunger signal that specifically promotes wakefulness through neurons expressing cryptochrome, without affecting feeding behavior, indicating functional segregation in NPF network regulation of sleep and feeding (Chung et al., 2017). AstA, secreted by enteroendocrine cells and neurons, promotes sleep by inhibiting visually guided movement (through targeting helicon cells) and regulating energy metabolism (through activation of adipokinetic hormone (Akh), a glucagon-like hormone), with its activity modulated by the circadian output signal PDF (Chen et al., 2016). Notably, the NPF/NPF receptor system exhibits male-specific sleep-promoting effects in *Drosophila*, potentially linked to sexually dimorphic expression patterns in D1 neurons (He et al., 2013).

Other neuropeptide signaling pathways, including SIFamide (SIFa), FMRFamide, and Leucokinin, integrate environmental stress, metabolism, and sleep regulation (Zhao et al., 2024; Lenz et al., 2015). SIFamide and its receptor (SIFR) promote sleep through a conserved neuropeptide signaling pathway, with expression in neurons localized to the pars intercerebralis (PI) of the central brain; activation of these neurons increases sleep duration and exhibits sexual dimorphism (Park et al., 2014; Huang et al., 2021). Activation of SIFa neurons depends not only on SIFa itself but may involve the release of other neurotransmitters, indicating the complexity of its sleep regulatory mechanisms (Huang et al., 2021). Under stress conditions, *Drosophila* sleep regulation involves the FMRFamide neuropeptide signaling pathway. This pathway promotes recovery sleep following heat stress through the FMRFamide receptor (FR), analogous to the function of FLP-13 neuropeptides in *Caenorhabditis elegans* (Lenz et al., 2015). This pathway differs from sleep regulation mechanisms involved in immune stress (such as infection), which depend on the NFκB transcription factor Relish, suggesting that different stressors modulate sleep through independent mechanisms (Lenz et al., 2015). The *translin (trsn)* gene, functionally localized in neurons secreting the Leucokinin neuropeptide, mediates sleep suppression during starvation (Murakami et al., 2016).

### 3.4 Ion channel signaling-related genes

Different subtypes of calcium channel-related genes in *Drosophila* exhibit diverse regulatory patterns. Sleep deprivation induces accumulation of the presynaptic protein Bruchpilot (BRP) in the MB and upregulates expression of *dSyd-1* and *Cacophony* (*cac*, the *Drosophila* Cav2 channel homolog), suggesting calcium channels may participate in sleep homeostasis regulation through synaptic plasticity (Weiss and Donlea, 2021). Deletion of the *cac* gene disrupts sleep-wake rhythmicity, manifesting as reduced nocturnal sleep, hyperactivity, and circadian rhythm disturbances—phenotypes resembling symptoms in schizophrenia patients (Hidalgo et al., 2021). MB-specific knockout of *cac* results in short- and medium-term memory deficits accompanied by decreased presynaptic calcium transients, indicating that Cav2 channels influence sleep and cognitive functions by modulating neuronal calcium signaling (Hidalgo et al., 2021). The *Drosophila* T-type calcium channel gene *Ca-*α*1T* (Cav3 homolog) functions differently from its mammalian counterpart. *Ca-*α*1T* deletion mutants exhibit increased sleep duration, particularly during subjective daytime under constant darkness conditions, suggesting that T-type channels in *Drosophila* may regulate sleep-wake balance by promoting wakefulness rather than stabilizing sleep (Jeong et al., 2015).

Voltage-gated channel-related genes in *Drosophila* primarily include *Shaker (Sh), Hyperkinetic (Hk), sleepless (sss)*, and *quiver (qvr)*, which influence sleep behavior by modulating potassium channel activity, neuronal excitability, and synaptic transmission. Shaker encodes a voltage-gated potassium channel; its loss-of-function mutants (such as *minisleep, mns*) exhibit significantly reduced sleep duration, impaired recovery sleep following sleep deprivation, and shortened lifespan (Cirelli et al., 2005; Kempf et al., 2019). Hyperkinetic, functioning as the β-regulatory subunit of Shaker, also leads to decreased sleep and memory deficits when mutated, further confirming the role of potassium currents in sleep maintenance (Bushey et al., 2007). *sleepless (sss)* encodes a Ly6/neurotoxin family protein that promotes sleep by directly binding to Shaker channels and regulating their expression levels, kinetic properties, and membrane localization; *sss* mutants display reduced sleep time and lowered arousal thresholds (Koh et al., 2008; Wu et al., 2010, 2014). Additionally, *sss* further modulates sleep by inhibiting nicotinic acetylcholine receptor (nAChR) activity, thereby reducing synaptic transmission (Wu et al., 2014). *quiver (qvr)*, an allele of *sss*, influences neuronal excitability by extracellularly regulating the frequency response characteristics of Shaker channels (Wu et al., 2010; Wang and Wu, 2010). The interactions among these genes reveal the central position of potassium channel regulation, neuronal excitability, and synaptic plasticity in sleep homeostasis (Kempf et al., 2019). Furthermore, mitochondrial metabolites dynamically regulate Shaker channel activity through the NADPH redox state of *Hk*, linking energy metabolism with sleep requirements (Kempf et al., 2019).

Calcium-activated potassium channel-related genes and their regulatory networks in *Drosophila* participate in sleep regulation by integrating neuronal excitability, synaptic transmission, and neuroglia interactions. Mutation of the voltage-gated potassium channel Kv9.2 (encoded by *KCNS2*; hKv9.2-D379E) induces neuronal hyperexcitability in *Drosophila* models, characterized by increased inactivation of Shab channels (*Drosophila* Kv2 homologs) and elevated spontaneous firing frequency, concurrently triggering nocturnal activity and sleep disruption (Smith et al., 2018). Similarly, deficiency in the calcium-activated potassium channel *ORK1* (TREK-1 homolog) reduces sleep duration, while its overexpression increases sleep, demonstrating bidirectional sleep regulation through modulation of neuronal excitability (Zhang et al., 2017). Slowpoke binding protein (SLOB) and Na^+^/K^+^ ATPase β subunit (NaKβ) influence nocturnal rhythmic firing patterns by regulating membrane excitability and synaptic properties of circadian neurons (such as DN1p). Aging disrupts the subcellular localization of these molecules, leading to decreased sleep quality (Nguyen et al., 2022). The SLEEPLESS protein, encoded by *sleepless (sss)*, regulates sleep through a dual mechanism: upregulating the open probability of Shaker potassium channels to suppress neuronal excitability (Wu et al., 2010, 2014), while concurrently reducing synaptic transmission through direct antagonism of nicotinic acetylcholine receptors (nAChRs; Wu et al., 2014). Elevated expression of GABA transaminase (GABAT) in *sss* mutants results in decreased GABA levels, with enhanced GABAT activity in glial cells serving as a critical factor in sleep reduction in *sss* mutants (Chen et al., 2015). The kinetic properties of Shaker channels (such as activation velocity and C-type inactivation) are regulated by the SSS protein, with these properties more directly influencing sleep phenotypes than current amplitude (Dean et al., 2011). Structural analysis reveals that SSS interacts with potassium channels and nAChRs through its loop 2 domain, elucidating the molecular basis by which the Ly6 protein family regulates neural function (Wu et al., 2016). In Parkinson's disease models, Usp14 downregulation improves sleep and circadian rhythm deficits in *Pink1* mutant flies by enhancing proteasome activity (Favaro et al., 2024), while deficiency in Mbt/PAK4 kinase results in reduced dopaminergic neurons and sleep fragmentation (Pütz et al., 2021), suggesting that calcium-activated potassium channel-related genes may indirectly regulate sleep through dopaminergic pathways.

Hyperpolarization-activated cyclic nucleotide-gated channels (HCN or Ih) in *Drosophila melanogaster* participate in sleep and rhythmicity regulation by modulating dopaminergic signaling and electrical activity of clock neurons. Absence of Ih currents significantly affects dynamic dopamine fluctuations, disrupting sleep-activity patterns in *Drosophila*. Mutants lacking the *DmIh* gene exhibit abnormal dopamine levels, particularly excessive dopamine accumulation under dark conditions, while periodic dopamine fluctuations under light conditions depend on Ih currents, resulting in sleep fragmentation and circadian rhythm disorders (Gonzalo-Gomez et al., 2012). Behavioral analyses demonstrate that Ih channel mutants display shortened lifespan, altered chemical sensitivity, and reduced sleep time under light-dark cycles, indicating that these channels regulate behavior through multiple mechanisms (Chen and Wang, 2012). Electrophysiological studies reveal that Ih currents are essential for the high-frequency burst firing patterns of ventral lateral neurons (LNvs), a firing mode that promotes the release of pigment dispersing factor (PDF), thereby coordinating circadian behaviors and sleep (Fernandez-Chiappe et al., 2021).

Transient Receptor Potential (TRP) channel-related genes in *Drosophila* primarily regulate sleep by integrating environmental temperature, light signals, and neural circuit activity. TrpA1, a temperature-sensitive TRP channel, delays the onset of daytime siesta in high-temperature environments, a phenomenon termed “Prolonged Morning Wakefulness” (PMW; Lamaze et al., 2017). *TrpA1*-expressing neurons modulate temperature-dependent sleep plasticity through synaptic connections with DN1p circadian neurons (Lamaze et al., 2017). TrpA1 can be utilized for remote thermal activation of specific neurons (such as dFB projection neurons) to induce sleep, thereby promoting long-term memory consolidation (Donlea et al., 2011). Under sleep deprivation conditions, activation of TrpA1 in the gut promotes Allatostatin A (AstA) release through reactive oxygen species (ROS) accumulation, subsequently regulating metabolic dysfunction (Li et al., 2023b). Another investigation revealed that calcium (Ca^2+^) signaling in astrocytes, dependent on L-type calcium channels (potentially in coordination with TRP channels), encodes sleep need and regulates sleep homeostasis through the release of the neuropeptide Spätzle (Blum et al., 2021). Additionally, within the temperature-sensitive AC-DN1p-PI neural circuit, DN1p neurons integrate temperature inputs to promote wakefulness via CNMa signaling pathway (Jin et al., 2021).

### 3.5 Transcription regulatory genes

Various transcription factors and their associated genes participate in sleep regulation through multi-layered control systems, from circadian oscillations to neuropeptide release, hormonal signaling to neuronal excitability. The *Tango10* gene functions as an E3 ubiquitin ligase adaptor within pigment dispersing factor (PDF)-expressing pacemaker neurons, regulating rhythmic neuropeptide release. *Tango10* forms a complex with CULLIN 3 (CUL3) ubiquitin ligase to collectively regulate PDF stability (Lee et al., 2021). Tango10 mutation leads to abnormal PDF accumulation at nerve terminals, even in the absence of a functional core circadian clock (such as in *timeless* gene deficiency). Electrophysiological recordings demonstrate enhanced spontaneous firing activity in *Tango10* mutant neurons, potentially related to reduced voltage-gated Shaker-like potassium currents. This suggests the Tango10/Cul3 pathway transforms molecular oscillations of the core circadian clock into rhythmic neuropeptide release outputs, regulating *Drosophila* sleep-wake behavioral rhythms.

The circadian gene network plays a foundational role in sleep regulation, with rhythmic expression of genes such as *timeless (tim)* and *vrille (vri)* being particularly important and subject to regulation by microRNAs including *miR-375* (Xia et al., 2020). In *Clock (Clk)* mutant flies, this regulatory network is disrupted, leading to abnormal sleep patterns. With *Drosophila* aging, transcriptional oscillations of *tim* and *period (per)* significantly attenuate, closely associated with sleep fragmentation and activity rhythm disorders (Rakshit et al., 2012). In Huntington's disease (HD) models, abnormal expression of *tim* and *vri* further results in decreased sleep and prolonged sleep latency (Faragó et al., 2019). *tim* also participates in rhythmic regulation of steroid hormone synthesis through insulin and prothoracicotropic hormone (PTTH) signaling pathways, indirectly influencing development and metabolism-related sleep behaviors (Di Cara and King-Jones, 2016).

The transcription factor ATF-2 (dATF-2) is predominantly expressed in l-LNvs and s-LNvs, with only l-LNvs being specifically labeled by antibodies recognizing phosphorylated dATF-2. RNA interference-mediated knockdown of *dATF-2* results in decreased sleep duration, while overexpression increases sleep time, primarily by affecting sleep bout length (Shimizu et al., 2008). dATF-2 also participates in regulating post-sleep-deprivation rebound and arousal threshold modulation, with phosphorylation levels higher in the morning than at night and activatable through the dp38 pathway by forced locomotion, suggesting dATF-2 may function as a regulatory factor connecting locomotion and sleep.

*CncC* (the mammalian Nrf2 homolog) significantly influences sleep by regulating redox homeostasis. Overexpression of *CncC* or inhibition of its negative regulator Keap1 alters *Drosophila* sleep patterns, and antioxidant supplementation can mimic the effects of enhanced *CncC* signaling (Spiers et al., 2019). Changes in *heme oxygenase (ho)* expression levels in neurons and glial cells affect adult *Drosophila* sleep patterns, with effects dependent on gene expression timing, cell type, and fly sex and age (Bilska et al., 2023).

Ecdysone and its receptor EcR, together with its heterodimeric partner Ultraspiracle (usp, an RXR homolog), collectively respond to hormonal signals in sleep regulation. EcR and its downstream nuclear receptor E75 are expressed in glial cells (particularly cortex glia), regulating sleep rhythmicity and total amount through lipid metabolism modulation (such as lipid droplet mobilization; Li et al., 2023a). Exogenous ecdysone treatment promotes sleep in a dose-dependent manner, primarily by extending the duration of both sleep and wake bouts (Ishimoto and Kitamoto, 2010). Mutants of ecdysone synthesis genes exhibit “short sleep” phenotypes, which can be alleviated by supplementation with 20-hydroxyecdysone (20E) during adulthood (Ishimoto and Kitamoto, 2010). Endogenous ecdysone levels increase following sleep deprivation, and mutants with ecdysone signaling defects display reduced sleep rebound, indicating ecdysone also participates in sleep homeostasis regulation.

*MEF2C (dMEF2)* function in *Drosophila* is closely associated with sleep and activity. Knockdown of *dMEF2* in dopaminergic neurons significantly increases locomotor activity and reduces sleep, consistent with hyperactivity and sleep disorder phenotypes related to human attention deficit hyperactivity disorder (ADHD; Klein et al., 2020). In mammals, MEF2 family genes also participate in sleep and circadian rhythm regulation; for example, MEF2D deficiency in mice alters free-running rhythmic periods and sleep patterns (Mohawk et al., 2019).

*zfh1* and several other genes (including *bin3, blot, CG42389, kirre, slim*, and *VAChT*) simultaneously regulate ovarian tubule number and sleep behavior, revealing their dual roles in reproduction and sleep regulation (Lobell et al., 2017). These genes exhibit pleiotropy, independently affecting sleep parameters and ovarian tubule numbers, with low linkage disequilibrium between their polymorphisms, suggesting they may regulate sleep and reproductive functions through different mechanisms.

*TfAP-2*, a member of the AP-2 transcription factor family, plays a crucial regulatory role in nighttime sleep. Specific knockdown of *TfAP-2* in the nervous system results in almost complete disappearance of nighttime sleep, while daytime sleep remains unaffected (Kucherenko et al., 2016). Additionally, *TfAP-2* insufficiency affects nervous system development, and conditional knockdown of *TfAP-2* in adult flies also leads to mild sleep phenotypes, suggesting *TfAP-2* functions not only during larval stages but also continuously regulates sleep in differentiated neurons.

High sleep pressure rapidly reprograms *Drosophila* wake-promoting neurons—l-LNvs—to express pigment dispersing factor receptor (PDFR). This receptor reconfiguration depends on two DA receptors and the transcriptional regulator nejire (CREBBP), activated through the cAMP signaling pathway, thereby enhancing waking behavior and improving early mating success (Klose and Shaw, 2021). This mechanism suggests that PDFR re-expression can reshape neural circuit function, adapting fly sleep levels to environmental demands.

The *trp*γ gene (homologous to human TRPC6) plays an important role in sleep regulation in *Drosophila*. Loss-of-function mutations in *trp*γ result in behavioral deficits resembling autism spectrum disorder (ASD), including disruption of sleep homeostasis, without affecting circadian control of sleep (Palacios-Muñoz et al., 2022). These sleep defects exhibit sex and age dependence, with symptoms more severe in certain sexes and progressively worsening with age. Notably, the TRPC6 agonist hyperforin (the primary active component of St. John's wort extract) significantly alleviates sleep defects in *trp*γ mutant flies, suggesting the TRPC6 pathway may represent a potential target for sleep regulation.

### 3.6 Genes related to RNA/protein modification

RNA editing, small RNA pathways, chromatin remodeling, tRNA modifications, and long non-coding RNAs influence *Drosophila* sleep behavior through multi-layered regulatory networks. Adenosine Deaminase Acting on RNA (ADAR) catalyzes the conversion of adenosine to inosine during RNA editing. In *Drosophila, ADAR* gene defects result in increased sleep. In *ADAR* mutants, vesicular glutamate transporter expression is upregulated, NMDA receptors are excessively activated, and the reserve pool of glutamatergic synaptic vesicles is selectively expanded. These alterations enable synapses to maintain sustained neurotransmitter release under conditions that would normally induce synaptic depression, leading to increased sleep pressure (Robinson et al., 2016).

microRNA-276a (*miR-276a*) expression is directly regulated by core circadian transcription factors CLOCK/CYCLE (CLK/CYC), with its promoter located in the 8th fragment (aFrag8) of pre-miR-276a, which can be activated by CLK/CYC. Functional experiments demonstrate that *miR-276a* loss-of-function significantly increases daytime and nighttime sleep duration in *Drosophila*, while gain-of-function reduces sleep, indicating its negative regulatory role in sleep. *miR-276a* is widely expressed in mushroom bodies (MB), pars intercerebralis (PI), and certain clock neurons (such as LNds), with *timeless (tim)*-expressing neurons being particularly critical for sleep regulation. *miR-276a* regulates sleep behavior by suppressing downstream target genes *tim* and *neuropeptide F receptor 1* (*npfr1*). In wild-type flies (w1118), *miR-276a* expression exhibits rhythmic oscillation, which disappears in clk loss-of-function mutants (*clkjrk*), further confirming CLK/CYC regulation of its expression (Zhang et al., 2021a).

miR-276b influences sleep behavior by targeting *tim, npfr1*, and *dopamine receptor 1 (DopR1)* genes. Research shows that flies lacking *miR-276b* exhibit significantly increased sleep duration, while flies overexpressing *miR-276b* display reduced sleep. The promoter region of miR-276b responds to CLOCK protein, suggesting its involvement in circadian regulation through the CLK/CYC-TIM/PER negative feedback loop. *miR-276b* is widely expressed in clock neurons, mushroom bodies, and fan-shaped bodies in the *Drosophila* brain, and its overexpression significantly reduces sleep duration (Zhang et al., 2021b).

miR-92a exhibits rhythmic expression in pigment dispersing factor (PDF) neurons of *Drosophila* and regulates PDF neuron excitability by inhibiting Sirtuin 2 (SIRT2) expression, thereby influencing circadian rhythms and sleep behavior (Chen and Rosbash, 2017). A miRNA sponge screening study identified 25 miRNAs that regulate baseline sleep, among which miR-92a/92b/310 family members display similar sleep regulatory functions, while let-7 miRNA regulates sleep homeostasis through dual developmental and adult actions in the mushroom bodies (Goodwin et al., 2018).

microRNA bantam regulates early night sleep in *Drosophila* through specific neuronal subpopulations. Bantam promotes early night sleep by inhibiting the activity of γ5β′2a/β′ 2mp/β′2mp bilateral mushroom body output neurons (MBONs). These glutamatergic neurons integrate environmental information and regulate behavior. Calcium imaging experiments reveal that bantam significantly suppresses these MBONs' activity during early night but not during daytime. Blocking synaptic transmission in these MBONs rescues sleep phenotypes caused by bantam knockdown, indicating that bantam promotes sleep by inhibiting MBON excitability. Through RNA sequencing analysis, Kelch protein and CCHamide-2 receptor were identified as potential downstream effector molecules of bantam (Hobin et al., 2022).

The chromatin remodeling factor Imitation SWItch/SNF (ISWI) participates in adult *Drosophila* sleep regulation by modulating neurogenesis and brain region formation during development. ISWI loss-of-function results in reduced sleep during adulthood, accompanied by circadian rhythm disruption, memory deficits, and abnormal social behavior (Gong et al., 2021). ISWI exhibits cell type-specific and developmental stage-specific functions; for example, its expression in type I neuroblasts is crucial for adult sleep and the formation of learning-related brain regions. Human ISWI homologs SMARCA1 and SMARCA5 can partially rescue phenotypes caused by ISWI deficiency in *Drosophila*, but SMARCA5 variants from neurodevelopmental disorder patients fail to restore sleep defects, highlighting the critical role of chromatin remodeling mechanisms in the development of sleep neural circuits (Gong et al., 2021).

*Tip60*, a histone acetyltransferase (HAT), functions in *Drosophila* sleep regulation through epigenetic mechanisms. *Tip60* interacts with the intracellular domain of amyloid precursor protein (APP), an Alzheimer's disease-associated protein, regulating axonal growth of sLNv and expression of the neuropeptide PDF, thereby influencing sleep-wake cycles. Under conditions of *Tip60* HAT activity deficiency, APP's neurodegenerative conditions lead to reduced PDF expression, retraction of sLNv synaptic structures, and subsequent disruption of sleep-wake rhythms. *Tip60* overexpression completely rescues these sleep disorders by promoting overgrowth of sLNv synaptic terminals and increasing PDF levels, indicating Tip0's neuroprotective role in these processes (Pirooznia et al., 2012; Pirooznia and Elefant, 2013).

Elongator Protein 3 (Elp3), the highly conserved catalytic subunit of the Elongator complex, possesses multiple functions in neuronal nuclei and cytoplasm, including regulation of neuron-motility related genes through epigenetic mechanisms and influence on axonal branching and cortical neuron migration via α-tubulin acetylation (Singh et al., 2010). Specific reduction of ELP3 expression during *Drosophila* nervous system development results in hyperactivity and sleep loss phenotypes in adult flies. Additionally, significant increases in synaptic bouton numbers and extensions in axonal length and branching are observed at larval neuromuscular junctions, accompanied by dysregulation of genes associated with these processes (Singh et al., 2010).

The long non-coding RNA (lncRNA) yellow-achaete intergenic RNA (*yar*) is highly conserved across multiple *Drosophila* species, with its promoter region sequence and expression timing maintained consistently in *Drosophila melanogaster* and *D. virilis*. By constructing yar deletion mutants, researchers discovered significant sleep behavior abnormalities: reduced and fragmented nighttime sleep, along with impaired recovery capability following sleep deprivation. These phenotypes can be completely restored by introducing *yar* transgenes, confirming yar's specific role in sleep regulation. Further research indicates that *yar* is a cytoplasm-localized lncRNA, suggesting it may influence sleep by regulating mRNA stability or translation processes (Soshnev et al., 2011).

TAR DNA-binding protein 43 (TDP-43) significantly disrupts sleep homeostasis through an Ataxin-2 (Atx2)-dependent metabolic dysregulation pathway. Expression of human TDP-43 in *Drosophila* causes severe sleep fragmentation, a phenotype significantly improved by *Atx2* gene knockdown. Brain transcriptome analysis reveals that Atx2 interference primarily regulates transcripts associated with small molecule metabolic signaling, particularly in the context of TDP-43 expression. Further screening identifies that among Atx2-regulated genes, those involved in metabolic pathways (such as glycogen metabolism-related genes) have inhibitory effects on TDP-43-induced sleep disorders. Additionally, rapamycin treatment or Atx2 knockdown not only alleviates sleep defects caused by TDP-43 but also improves glycogen metabolism dysregulation induced by TDP-43 (Perlegos et al., 2024).

### 3.7 Genes related to metabolism

*Drosophila* lipoprotein receptors LpR1 and LpR2, members of the low-density lipoprotein receptor family, mediate lipid uptake. LpR deficiency leads to abnormal sleep patterns in *Drosophila*, potentially associated with mushroom body developmental defects. The mushroom body represents a central nervous system structure in *Drosophila* involved in learning, memory, and sleep regulation. *In vitro* experiments demonstrate that mammalian Reelin protein enhances neuritic branching complexity in MB neurons, a process dependent on LpRs and Disabled (Dab). Although *Drosophila* lacks Reelin homologs, the long isoforms of LpRs can mediate Reelin internalization, suggesting that LpRs participate in neurodevelopment and functional regulation through conserved signaling pathways, influencing behaviors such as sleep (Rojo-Cortés et al., 2022).

The adipokinetic hormone-forkhead box O (AKH-FOXO) and insulin/insulin-like growth factor signaling/target of rapamycin (IIS/TOR) pathways contribute to sleep regulation in *Drosophila*. Starvation regulates sleep through the AKH-FOXO pathway, where deficiency of AKH and its receptor AKHR blocks starvation-induced dorsal projection extension of s-LNv, alleviating sleep suppression caused by food deprivation. FOXO, acting as a starvation-response factor, modulates neuronal synaptic plasticity, affecting s-LNv projections and sleep (He et al., 2020). Reduced IIS/TOR signaling network activity improves sleep fragmentation in aging *Drosophila*, with nighttime sleep continuity and consolidation dependent on S6 kinase (S6K) and attenuated dopaminergic signaling, while daytime activity is mediated through AKH, dFOXO, and octopaminergic signaling (Metaxakis et al., 2014).

Glial cells participate in *Drosophila* sleep and circadian rhythm regulation by modulating sphingolipid metabolism. Glucocerebrosidase 1b (GBA1b) regulates dynamic structural remodeling of circadian neurons through sphingolipid degradation. In *gba1b* mutants, sphingolipid accumulation causes lysosomal dysfunction, triggering protein aggregation that fluctuates with circadian rhythms and is regulated by neuronal activity, biological clock, and sleep. Sphingolipid biosynthesis and degradation are crucial for circadian remodeling of clock neurons (such as sLNvs), influencing rhythmic behavior and sleep patterns (Vaughen et al., 2022). *Lipid storage droplet-2 (Lsd2)* mutant flies exhibit deficient lipid accumulation capacity, weakened sleep homeostatic response, and unimpaired learning ability following sleep deprivation, indicating that Lsd2 maintains sleep homeostasis through lipid metabolism regulation (Thimgan et al., 2010). Ecdysone regulates sleep rhythmicity and quantity through ecdysone receptor (EcR) and E75 in glial cells, with *lsd2* mutants showing reduced response to the sleep-promoting effects of exogenous ecdysone (Li et al., 2023a).

The *Angiotensin-converting enzyme-related (ACER)* gene contributes to *Drosophila* nighttime sleep maintenance and integration of metabolism-sleep signaling pathways. Acer deletion mutants exhibit reduced nighttime sleep and increased sleep fragmentation, with ACER influencing sleep-wake balance through regulatory peptide cleavage. Acer mutation disrupts adaptive responses to nutritional changes, potentially related to abnormal IIS pathway function (Carhan et al., 2011; Glover et al., 2019). Sterol regulatory element-binding protein (SREBP) and Malic enzyme (Men) affect nighttime sleep by regulating the NADP+/NADPH cycle. Increased SREBP activity promotes wake-related gene transcription, disrupts the NADP+/NADPH ratio, and reduces nighttime sleep pressure, while reducing SREBP or Men activity can improve sleep defects (Mariano et al., 2023). Insomnia model flies demonstrate that sleep deprivation leads to differential expression of genes related to metabolism, neuronal activity, and sensory perception, associated with sleep disorders, cognitive impairment, and metabolic abnormalities (Seugnet et al., 2009).

### 3.8 Genes related to synapse development

The *Drosophila Homer* gene maintains sleep stability by regulating synaptic plasticity of metabotropic glutamate receptors (mGluRs). Specific knockdown of *Homer* in the fly brain results in reduced sleep, and Homer protein binds to *Drosophila*'s sole metabotropic glutamate receptor, DmGluRA (Ly et al., 2020). This interaction is crucial for sleep promotion, as disruption of the Homer binding site (PPXXF sequence) on DmGluRA using CRISPR/Cas9 technology significantly reduces Homer-DmGluRA interaction, leading to shortened sleep duration (Ly et al., 2020). Homer protein expression is upregulated during sleep, while its immediate early gene form, *Homer1a*, shows increased expression during wakefulness, indicating dynamic regulatory roles of Homer across different sleep-wake states (Naidoo et al., 2012). In aging flies, expression levels of *mGluR* and its binding scaffold proteins Homer and Shank decline, closely associated with age-related sleep disorders and memory impairment (Hou et al., 2023). Overexpression of *mGluR* in neurons improves sleep in both young and aging flies, suggesting a conserved role of the Homer-mGluR signaling pathway in maintaining sleep homeostasis (Hou et al., 2023).

*Drosophila* Neuroligin 4 (DNlg4) participates in sleep regulation by modulating GABAergic neurotransmission. DNlg4 is highly expressed in l-LNvs, and l-LNv-specific expression of DNlg4 is critical for sleep regulation (Li et al., 2013). In *dnlg4* mutants, GABA transmission function in l-LNvs is impaired, leading to abnormal sleep phenotypes, but genetic restoration of GABA transmission can rescue these sleep defects (Li et al., 2013). Further investigation reveals significantly reduced GABA_A_ receptor Resistant to Dieldrin (RDL) clusters in *dnlg4* mutant flies, with DNlg4 interacting with RDL receptors *in vivo* (Li et al., 2013).

The *Drosophila* Leukocyte-antigen-related-like (*Lar*) gene contributes to sleep regulation by acting on local MB neural circuits (Draper et al., 2024). Research indicates that a sleep-regulating protein containing Ig domains, Noktochor (NKT), is secreted by mushroom body α′/β′ neurons and acts on other MB neuron subtypes (Draper et al., 2024). When membrane-anchored NKT (tNkt) is expressed in pan-neurons or broad MB neurons, sleep duration decreases, similar to the phenotype of NKT deletion mutants, suggesting tNkt might block endogenous NKT receptor function (Draper et al., 2024). The Lar receptor likely mediates NKT's effects: knockdown of Lar in MB increases sleep, while Lar overexpression reduces sleep, indicating a wake-promoting function of the Lar receptor (Draper et al., 2024). Surprisingly, selective expression of *tNkt* or *Lar* knockdown in MB wake-promoting neurons increases sleep duration, suggesting NKT not only acts on sleep-promoting neurons but may also regulate sleep by modulating wake-promoting neuron activity (Draper et al., 2024).

The *Drosophila Ten-a* gene exerts sleep regulatory functions in the central complex (Cheng et al., 2013). Mutation of the *Ten-a* gene (*cbd* mutant) causes morphological abnormalities in the central complex, particularly fusion defects of the FB primordium (Cheng et al., 2013). Ten-a loss-of-function may prevent normal retraction of C767-Gal4-marked interhemispheric connections, disrupting fusion of the FB primordium, ultimately affecting central complex neural circuit formation and undermining the neural basis of sleep regulation (Cheng et al., 2013).

In *Drosophila*, the Mushroom bodies tiny (*Mbt*) gene, a homolog of p21-activated kinase 4 (*PAK4*), has been implicated in Parkinson's disease (PD)-related phenotypes and sleep regulation (Pütz et al., 2021). *mbt* mutant flies exhibit age-dependent motor deficits, shortened lifespan, and sleep fragmentation, resembling PD symptoms (Pütz et al., 2021). Sleep fragmentation, a non-motor symptom of PD, and the disrupted sleep architecture in *mbt* mutant flies further indicate this gene's important role in sleep homeostasis regulation (Pütz et al., 2021). *mbt* loss-of-function negatively impacts the number of dopaminergic neurons (PAM cluster), possibly due to neuronal precursor proliferation defects (Pütz et al., 2021). Notably, while age-dependent motor deficits do not coincide with further PAM neuron loss, Mbt deficiency in specific PAM subgroups directly leads to motor function impairment, and restoring Mbt expression in these neuronal subgroups extends lifespan, suggesting Mbt regulates sleep-wake behavior and longevity through dopaminergic neural circuits (Pütz et al., 2021).

### 3.9 Genes related to protein degradation

The ubiquitin-proteasome system-related genes that function through synaptic homeostasis, neuropeptide secretion, and dopaminergic pathways play roles in sleep regulation. The Cullin-3 (Cul3) ubiquitin ligase complex and its BTB domain adaptor proteins (including *insomniac* and *BTBD9*) regulate sleep homeostasis and wakefulness through protein degradation (Pfeiffenberger and Allada, 2012; Stavropoulos and Young, 2011). Mutations in the *insomniac (inc)* gene reduce total sleep time by approximately 10 hours and impair sleep consolidation and homeostatic recovery (Pfeiffenberger and Allada, 2012; Stavropoulos and Young, 2011). These sleep deficits can be rescued by inhibiting tyrosine hydroxylase, the rate-limiting enzyme in dopamine synthesis (Pfeiffenberger and Allada, 2012). Both *Cul3* and *inc* show developmental expression in neurons that persistently affects adult sleep function (Li et al., 2021), with *inc* mutations causing structural defects and overproduction of mushroom body neurons (Li et al., 2021). BTBD9, the *Drosophila* homolog of a human restless legs syndrome risk gene, influences sleep fragmentation by disrupting iron regulatory protein accumulation and reducing tyrosine hydroxylase activity (Freeman et al., 2013).

The fragile X messenger ribonucleoprotein 1 (*Fmr1*) gene plays a role in sleep-dependent synaptic homeostasis (Bushey et al., 2011). Wakefulness increases synaptic size and number, while sleep requires *Fmr1* for synaptic renormalization (Bushey et al., 2011). Fmr1 deficiency also causes arrhythmic locomotor activity under constant darkness (Inoue et al., 2002). The F-box protein Fbxl4 regulates sleep by rhythmically degrading GABA_A_ receptors (Grover et al., 2022). Its transcription in wake-promoting l-LNvs is CLOCK-dependent (Grover et al., 2022), reducing GABA sensitivity to promote wakefulness (Grover et al., 2022).

Parkinson's disease-related genes *parkin* and *pink1* affect circadian rhythms and sleep patterns (Valadas et al., 2018). These mutations increase endoplasmic reticulum-mitochondria contact sites (Valadas et al., 2018), leading to phosphatidylserine depletion from the ER, impaired neuropeptide vesicle production, and sleep disturbances that can be rescued by phosphatidylserine supplementation (Valadas et al., 2018).

### 3.10 Genes related to immune/stress response

Dorsal-related immunity factor (*Dif* ) functions as a transcription factor in the Toll pathway, regulating both baseline and recovery sleep in the central nervous system. *Dif* mutants exhibit reduced daily sleep and impaired recovery sleep. Its function primarily depends on brain expression, particularly in the pars intercerebralis region. *Dif* promotes deep sleep by inducing expression of the antimicrobial peptide *nemuri* (O'Hara et al., 2024). Peptidoglycan recognition protein LE (PGRP-LE) recognizes bacterial peptidoglycan and activates the immune deficiency (IMD) pathway. Gut microbiota (such as *Lactiplantibacillus plantarum*) influence sleep by activating insulin-producing neurons through peptidoglycan-PGRP-LE signaling (Xu et al., 2023).

*Dif* and *Relish* exhibit redundant functions in post-infection sleep regulation. Single mutants (lacking either *Dif* or *Relish*) retain responsiveness to sleep deprivation, but double mutants completely lose enhanced post-infection sleep and improved survival phenotypes, indicating that the Toll pathway coordinates immunity and sleep through NFκB-dependent mechanisms (Kuo and Williams, 2014).

*Relish* serves as the NF-κB transcription factor downstream of the IMD pathway. Gram-negative bacterial infection or sterile injury activates *Relish* through the IMD pathway, significantly increasing sleep in *Drosophila*, particularly during morning hours—a process dependent on the circadian clock gene *period*. Relish functions predominantly in the fat body. Relish null mutants fail to induce increased sleep following infection or injury, while transgenic expression of *Relish* in the fat body restores sleep responses (Kuo et al., 2010). Different stressors induce sleep through distinct mechanisms. Heat stress-induced sleep does not depend on *Relish* but is mediated by FMRFamide neuropeptide and its receptor FR. This pathway promotes recovery sleep across various stress conditions (Dissel et al., 2015).

*IM33* is an immunity-related gene whose mammalian homolog is secretory leukocyte protease inhibitor (SLPI). *IM33* expression is upregulated in aging flies, and its deletion in glial cells shortens lifespan by altering intestinal reactive oxygen species levels and microbiota composition (including increased *Lactiplantibacillus plantarum* abundance). Dysbiosis leads to sleep fragmentation by activating insulin-producing cells in the brain, through a mechanism involving DAP-type peptidoglycan produced by *L. plantarum* binding to the PGRP-LE receptor. *IM33* functions within the glia-microbiota-neuron axis, connecting neuroinflammation, dysbiosis, and sleep deterioration during aging (Xu et al., 2023).

Heat shock protein 83 (Hsp83) is a molecular chaperone involved in protein folding and stress responses. Flies carrying the *Hsp83* mutation (*Hsp*^8308445^) exhibit excessive homeostatic sleep responses and die following sleep deprivation, confirming the critical role of HSP family genes in maintaining sleep homeostasis. Period gene mutant (*cyc*^01^) flies display significant sleep rebound and mortality after 10 hours of sleep deprivation but demonstrate stronger resistance to other stressors. *cyc*^01^ mutants show reduced heat shock gene expression levels following sleep deprivation, and pre-activation of these heat shock genes can rescue sleep deprivation-induced mortality (Shaw et al., 2002).

*nemuri* is a sleep-inducing gene encoding the antimicrobial peptide NEMURI. NEMURI promotes prolonged sleep, enhances waking resistance, and improves survival rates following infection. This gene is specifically activated when sleep need increases (during sleep deprivation or bacterial infection) and targets sleep-promoting dFB neurons. When *Dif* is mutated, nemuri induction following sleep deprivation is significantly reduced, while pan-neuronal overexpression of *nemuri* partially rescues the sleep phenotype in *Dif* mutants (O'Hara et al., 2024; Toda et al., 2019).

### 3.11 Genes related to circadian rhythm

The *Clock (Clk)* gene encodes a transcription factor that is a pivotal component of the positive regulation of the circadian clock. Clk forms a heterodimer with cycle (cyc) to activate the transcription of *period (per)* and *timeless (tim)*. *Clk* mutants exhibit disrupted sleep rhythms and suppressed sleep under starvation conditions (Keene et al., 2010). These mutants also show reduced exercise endurance, which can be restored with octopamine (OA) supplementation (Safdar and Wessells, 2023). The expression of *Clk* in specific clock neurons, such as PDF neurons, is crucial for maintaining dopaminergic neuron survival and preventing premature motor decline (Vaccaro et al., 2017).

*Cycle (cyc)* forms a heterodimer with *Clk* to co-activate the transcription of *per* and *tim*. The *cyc*^01^ mutant is extremely sensitive to sleep deprivation, displaying severe sleep rebound and lethality, which is associated with reduced expression of heat shock proteins (HSPs; Shaw et al., 2002). Male *cyc*^01^ mutant flies exhibit diminished sleep compensation responses and shortened lifespan, demonstrating sexual dimorphism (Hendricks et al., 2003). *cyc* mutants also show suppressed sleep under starvation conditions (Keene et al., 2010).

*Period (per)* encodes the PER protein, which, when complexed with TIM, inhibits the activity of CLK/CYC. Mutations in *per* lead to sleep fragmentation and rhythm disorders (Shaw et al., 2002). During aging, the transcriptional oscillation of per weakens, resulting in the attenuation of sleep rhythms (Rakshit et al., 2012). *per* mutants display significant endurance decline (Rakshit et al., 2013).

*Timeless (tim)* encodes the TIM protein, which, upon binding to PER, enters the nucleus to inhibit CLK/CYC. tim mutations cause sleep fragmentation and rhythm disorders, resembling the phenotype of familial advanced sleep phase syndrome (FASPS) in humans (Cai et al., 2021). The phosphorylation state of TIM regulates the nuclear-cytoplasmic shuttling of the PER-TIM heterodimer, thus modulating the periodicity of the circadian clock (Cai et al., 2021). *miR-375* affects sleep duration and rhythm by targeting the 3′UTR of tim (Wang et al., 2020).

*Cryptochrome (cry)* encodes a blue light receptor that mediates the degradation of TIM protein in response to light signals, synchronizing the circadian clock with external light-dark cycles. As a blue light/ultraviolet light sensor, CRY is involved in the magnetoreception ability of *Drosophila* and mediates the improvement of sleep quality by magnetic field exposure. Exposure to a 0.4–0.6 mT magnetic field significantly improves the quality of nighttime sleep in wild-type flies, but this effect is abolished in *cry* mutants (*cryb*; Kawasaki et al., 2023). The interaction between CRY and actin enhances the light sensitivity of the fly's compound eyes, thereby influencing the activity of clock neurons through phototransduction pathways (Schlichting et al., 2018). Under oxidative stress, CRY regulates the rhythmic expression of oxidative stress markers by maintaining the periodicity of the circadian clock, while *cryb* mutants show increased sensitivity to oxidative stress and rhythm disorders (Subramanian et al., 2014).

*Jetlag (jet)* participates in the light-dependent degradation of TIM protein, regulating the photic sensitivity of the circadian clock. Different hypnotic drugs, such as phenobarbital and pentobarbital, significantly alter the total sleep time in *Drosophila*, whereas melatonin primarily shortens sleep latency (Wang et al., 2020). Herbal extracts, such as Panax notoginseng and Withania somnifera, have a significant impact on total sleep time (Wang et al., 2020).

*Nocturnin (nocte)* affects the metabolic regulation of the circadian clock and may be related to RNA stability or translational control. nocte1 mutant flies can normally synchronize to light-dark cycles at constant temperature but exhibit synchronization defects when both light-dark and temperature cycles are present (Chen et al., 2018). The *nocte1* mutants show altered patterns of daytime napping, indicating that the *nocte* gene regulates sleep behavior by influencing the temperature input pathway of specific clock neurons (Chen et al., 2018).

*Dyschronic (dysc)* affects the development or function of clock neurons. Neurosystem-specific RNA interference reveals that dysc significantly impacts sleep phenotypes (Smith and Macdonald, 2020). A multiple-parent QTL mapping study confirms that dysc, along with Dopa decarboxylase and timeless, has a moderate effect on sleep phenotypes (Smith and Macdonald, 2020).

Neuropeptides such as PDF and ITP are key output signals of clock neurons, coordinating activity rhythms with sleep (Kawasaki et al., 2023). PDF influences sleep-wake transitions by regulating the firing frequency of lateral neurons (LNvs), while ITP acts in concert with PDF (Kawasaki et al., 2023). The dopaminergic system regulates nocturnal hyperactivity through the D2 receptor (dD2R; Lee et al., 2013). microRNAs like miR-276a regulate sleep duration and rhythm by targeting genes such as *tim, npfr1*, and *DopR1* (Wang et al., 2020). The E3 ubiquitin ligase Tango10/Cul3 complex converts the oscillation of core clock molecules into neuropeptide release, maintaining behavioral rhythms (Lee et al., 2021).

The traditional view holds that the core molecular mechanism of circadian rhythms relies on the transcriptional regulation of the period and timeless genes by the transcription factors CLK/CYC, with their protein products, PER/TIM, providing negative feedback to inhibit the transcriptional activity of CLK/CYC, thus establishing a ~24-hour transcription-translation negative feedback oscillatory loop. However, recent research has revealed that regulatory elements located in the upstream untranslated region (uORFs) of the *clock* gene mRNA can modulate sleep duration and rhythmic cycles by influencing the translation efficiency of the CLOCK protein (Sun et al., 2025). This discovery introduces a new post-transcriptional mechanism for rhythmic regulation.

### 3.12 Genes related to developmental regulation

*Drosophila* sleep exhibits pronounced sexual dimorphism, with males displaying significantly longer midday sleep duration than females. Targeted expression studies of *transformer (tra)* and *tra2* genes reveal that the mushroom body and fat body play crucial roles in sex-specific sleep regulation (Khericha et al., 2016). Feminization of the mushroom body reduces male midday sleep, while sex conversion in the fat body also affects sleep patterns, indicating multi-tissue coordination in sleep regulation (Khericha et al., 2016). The juvenile hormone (JH) signaling pathway modulates sexually dimorphic sleep through its receptor germ cell-expressed (GCE), with enhanced JH function amplifying sex differences (increased sleep in males, decreased in females), while JH deficiency blurs these differences (Wu et al., 2018). The JH-GCE pathway regulates sleep patterns through sex differentiation-related genes (*fruitless* and *doublesex* in males, *sex-lethal, transformer*, and *doublesex* in females) and functions independently of the circadian clock (Wu et al., 2018).

Flight behavior and sleep need exhibit a negative regulatory relationship. Disruption of *Drosophila* flight capability (blocking wing expansion programs, genetic or mechanical wing perturbations) significantly increases sleep duration (Melnattur et al., 2020). A sleep regulatory pathway extends from wing-specific sensory neurons through projection neurons in the ventral nerve cord, ultimately connecting to central brain neurons (Melnattur et al., 2020). The neuropeptide bursicon and its receptor rickets are key molecules linking wing expansion and sleep regulation (Melnattur et al., 2020). Flight obstruction activates these sleep-promoting projection neurons, manifested as elevated intracellular calcium levels and increased synaptic numbers in their axonal projections (Melnattur et al., 2020).

Methoprene-tolerant (Met) regulates neuronal morphology and sleep behavior through glia-neuron interactions (Wu et al., 2021). Met expression in glial cells negatively regulates mushroom body β lobe fusion and positively maintains projection pruning of PDF neurons (small ventral lateral neurons, sLNvs), thereby influencing sleep (Wu et al., 2021). Met maintains nighttime sleep through the α/β lobes of the mushroom body in a development-independent manner (Wu et al., 2021).

Enhanced JH function amplifies sexually dimorphic sleep phenotypes, extending sleep duration in males while shortening it in females; JH loss-of-function causes feminization of male sleep and masculinization of female sleep (Wu et al., 2018). In *gce null* mutants, JH fails to restore sexually dimorphic sleep phenotypes (Wu et al., 2018). The JH-GCE pathway regulates sleep patterns through sex differentiation-related genes (*fruitless* and *doublesex* in males, *sex-lethal, transformer*, and *doublesex* in females; Wu et al., 2018). JH-induced sexual dimorphism in sleep relates to sleep drive and is independent of the circadian clock (Wu et al., 2018). JH may exert additional effects in male flies by antagonizing age-related sleep reduction (Wu et al., 2018).

### 3.13 Genes related to cellular transport/secretion

*Drosophila* Syndecan (*dSdc*) likely integrates metabolic and neural signaling pathways, as *dSdc* mutant flies exhibit significantly prolonged sleep duration (De Luca et al., 2010). These mutants simultaneously display reduced fat storage, decreased metabolic rate, and impaired mitochondrial respiratory function. Brain insulin-like peptide expression levels are diminished in *dSdc* mutants, potentially regulating sleep indirectly through effects on energy metabolism. Single nucleotide polymorphisms (SNPs) in human Syndecan family genes (such as *SDC4 rs4599*) show significant associations with sleep duration (De Luca et al., 2010).

Members of the LAT1-like amino acid transporter family, Juvenile hormone Inducible-21 (*JhI-21*) and minidiscs (*Mnd*), regulate sleep/wake cycles in dopaminergic neurons (Aboudhiaf et al., 2018). Downregulation of either *JhI-21* or *Mnd* in dopaminergic neurons leads to increased daily sleep and extended nighttime sleep duration. L-DOPA (dopamine precursor) intake influences sleep through a *JhI-21*-dependent mechanism, with *JhI-21* downregulation reducing fly sensitivity to L-DOPA-induced sleep reduction. *JhI-21* downregulation also attenuates sleep reduction effects caused by sustained activation of dopaminergic neurons. Changes in target of rapamycin (TOR) activity within dopaminergic neurons can modulate sleep/wake states (Aboudhiaf et al., 2018).

The *ebony* gene encodes a glial-specific neurotransmitter modifying enzyme responsible for metabolizing aminergic neurotransmitters including dopamine, histamine, and serotonin through β-alanine conjugation (Pantalia et al., 2023). Contrary to expectations, *ebony null* mutants exhibit significantly increased sleep duration. Tissue-specific knockdown experiments confirm that *ebony's* sleep regulatory function depends on glial cell activity. Aminergic neurotransmitter levels are paradoxically reduced in these mutants, suggesting that *ebony* may indirectly influence sleep-wake balance by maintaining neurotransmitter homeostasis (Pantalia et al., 2023).

### 3.14 Genes related to molecular chaperones

The *Drosophila* homolog of huntingtin (*dhtt/dHtt/Htt*) encodes a protein analogous to human huntingtin, implicated in Huntington's disease. Expression of mutant *huntingtin* (*mutHtt*) disrupts *Drosophila* sleep patterns, manifested as reduced sleep duration, sleep fragmentation, and prolonged sleep latency (Faragó et al., 2019). These sleep deficits correlate with aberrant circadian gene expression: extended expression timing of period and timeless, alongside reduced expression levels of *vrille* (Faragó et al., 2019). Huntington's disease model flies exhibit sleep and activity abnormalities in early adulthood, including difficulty initiating sleep, sleep fragmentation, and nocturnal hyperactivity, associated with abnormal activation of the protein kinase A/cAMP response element-binding protein (PKA/CREB) signaling pathway (Gonzales et al., 2016). Reducing PKA signaling significantly ameliorates sleep deficits and extends lifespan (Gonzales et al., 2016). Enhanced autophagy pathways (through overexpression of *Atg8a*) partially rescue mutHtt-induced sleep and circadian behavioral deficits, potentially related to enhanced synaptic output, despite persistent mutant protein aggregation (Sharma et al., 2023).

Mesencephalic astrocyte-derived neurotrophic factor (*Manf* ; *Drosophila* homolog: *DmManf* ) encodes a secreted endoplasmic reticulum (ER) stress response protein (Walkowicz et al., 2017). DmMANF is highly expressed in the *Drosophila* visual system, particularly in glial cells of the first optic neuropil (lamina). Downregulation of DmMANF in glial cells leads to degeneration of lamina epithelial glial cells, characterized by abnormal autophagosomal membrane structures originating from the ER, and affects recycling of the photoreceptor neurotransmitter histamine (Walkowicz et al., 2017). Silencing of DmMANF in neurons or glial cells alters daily activity/sleep patterns in flies, with decreased daytime activity and increased nighttime activity. Glial cell-specific DmMANF silencing also shortens fly lifespan (Walkowicz et al., 2017). Expression levels of DmMANF in astrocyte-like glia (AlGl) and ensheathing glia (EnGl) regulate *Drosophila* activity and sleep (Walkowicz et al., 2021). In AlGl, DmMANF overexpression causes structural alterations in clock neurons expressing PDF. DmMANF also participates in glial circadian regulation, as DmMANF silencing eliminates diurnal oscillations of sodium pump α-subunit expression in lamina epithelial glial cells (Walkowicz et al., 2021).

*translin (trsn)* is a highly conserved RNA/DNA binding protein that functions in starvation-induced sleep suppression (Murakami et al., 2016). *translin* mutants fail to suppress sleep under starvation conditions, yet energy stores, free glucose levels, and feeding behavior remain unaffected (Murakami et al., 2016). *translin* is broadly expressed in *Drosophila* head neurons, transcriptionally upregulated during starvation, and functionally localized to neurons producing the tachykinin family neuropeptide Leucokinin (Murakami et al., 2016). Post-starvation refeeding promotes increased sleep, an effect independent of translin or adipokinetic hormone mutations, suggesting that post-starvation sleep increases relate to feeding behavior itself rather than directly resulting from sleep loss during starvation (Regalado et al., 2017). *Drosophila* exhibit reduced metabolic rate (MR) during sleep, and starvation inhibits this normal sleep-associated metabolic rate change (Stahl et al., 2017). *translin* mutants display lower basal metabolic rates but still exhibit further metabolic rate decreases in response to starvation, indicating that metabolic rate and sleep duration regulation are genetically separable (Stahl et al., 2017).

### 3.15 Genes related to cell cycle regulation

Cyclin A (*CycA*), TARANIS (*TARA*), and Cyclin-dependent kinase 1 (*Cdk1*) form a critical regulatory network governing *Drosophila* sleep (Afonso D. J. et al., 2015). TARA, the *Drosophila* homolog of the Trip-Br (SERTAD) family of transcriptional coregulators, interacts with *CycA* through its conserved CycA-binding domain to promote sleep (Afonso D. J. S. et al., 2015). Loss-of-function *TARA* mutations result in dramatically reduced sleep quantity (approximately 60%), with its function dependent on cholinergic neurons (Afonso D. J. et al., 2015).

TARA promotes sleep by regulating CycA protein levels to inhibit Cdk1 activity, while excessive *Cdk1* activation antagonizes TARA and *CycA* action, promoting wakefulness (Rogulja and Young, 2012). Reducing *Cdk1* levels rescues the short-sleep phenotypes of *tara* and *CycA* mutants, confirming *Cdk1* as a downstream effector in the TARA-CycA pathway (Afonso D. J. et al., 2015; Rogulja and Young, 2012).

*CycA* is expressed in approximately 14 neurons within the pars lateralis (PL) region of the *Drosophila* brain, constituting a novel wake-promoting center functionally analogous to the mammalian hypothalamus (Rogulja and Young, 2012). Neuronal reduction of *CycA* delays sleep-wake transitions, increases wake episodes during sleep, and attenuates homeostatic responses following sleep deprivation, indicating *CycA's* critical role in sleep homeostasis maintenance (Rogulja and Young, 2012).

## 4 Conclusions

This review summarizes the progress in sleep mechanisms research in *Drosophila melanogaster* over the past two decades, from the perspectives of neural structures and gene regulation. As a model organism, the fruit fly exhibits sleep behaviors highly similar to those of mammals, and its relatively simple nervous system, combined with powerful genetic tools, makes it an ideal model for studying sleep mechanisms. Despite significant advancements in understanding the sleep regulation mechanisms in fruit flies over the past 20 years, some limitations have begun to surface with the development of new technologies and a deeper understanding of the field.

The fundamental dilemma in current *Drosophila* sleep research lies in the coexistence of prolific discoveries and complex, overlapping mechanisms. At the neural structural level, while numerous “sleep-related” neurons and brain regions have been identified, interpretations of their functions often fall into the trap of oversimplification. For instance, specific neurons or neurotransmitters (such as glutamate) are often arbitrarily classified as promoting sleep or wakefulness, neglecting the fact that their roles may be reversed or modulated depending on the specific receptors, neural microcircuits, and experimental conditions (such as light exposure). At the genetic regulation level, the situation is even more pronounced. Through reverse genetics and a “candidate gene” approach, nearly 200 “sleep genes” have been identified, but this lengthy and mixed list lacks a unified core molecular model, akin to those seen in circadian rhythm research. Most of these genes cause only minor fluctuations in total sleep duration, likely influencing sleep indirectly through metabolic or stress-related pathways. The true core homeostatic genes responsible for sleep pressure sensing and regulation remain buried within this list. Existing phenotype analyses that rely on a single metric—total sleep time—fail to differentiate between “core regulation” and “peripheral modulation,” which has become a bottleneck contributing to fragmented mechanistic understanding.

To overcome these limitations, there is a need to develop and adopt more refined and minimally disruptive standardized research paradigms. At the neural level, sleep should be analyzed across different depths and stages under controlled conditions, such as constant darkness and temperature, in combination with high-throughput video tracking and computational staging methods. Tools like optogenetics and chemogenetics should be employed to achieve spatiotemporal control of specific neural circuits, isolating environmental interference and revealing endogenous homeostatic signals. Additionally, connectomics should be used to verify and map the input and output connections of known sleep-related neurons throughout the nervous system. At the genetic level, the focus must shift beyond the reliance on “total sleep time” toward multidimensional phenotypic analyses that reflect core characteristics of sleep homeostasis, such as sleep pressure accumulation and rebound (Faville et al., 2015). Ultimately, through the integration of multidimensional data and the establishment of standardized research paradigms, we will gain a deeper understanding of the regulatory mechanisms underlying sleep in *Drosophila*.
